# Machine-Based Morphologic Analysis of Glioblastoma Using Whole-Slide Pathology Images Uncovers Clinically Relevant Molecular Correlates

**DOI:** 10.1371/journal.pone.0081049

**Published:** 2013-11-13

**Authors:** Jun Kong, Lee A. D. Cooper, Fusheng Wang, Jingjing Gao, George Teodoro, Lisa Scarpace, Tom Mikkelsen, Matthew J. Schniederjan, Carlos S. Moreno, Joel H. Saltz, Daniel J. Brat

**Affiliations:** 1 Center for Comprehensive Informatics, Emory University, Atlanta, Georgia, United States of America; 2 Department of Biomedical Informatics, Emory University, Atlanta, Georgia, United States of America; 3 College of Computing, Georgia Institute of Technology, Atlanta, Georgia, United States of America; 4 Department of Neurology, Henry Ford Hospital, Detroit, Michigan, United States of America; 5 Department of Pathology and Laboratory Medicine, Emory University, Atlanta, Georgia, United States of America; 6 Winship Cancer Institute, Emory University, Atlanta, Georgia, United States of America; Max Delbrück Center for Molecular Medicine, Germany

## Abstract

Pathologic review of tumor morphology in histologic sections is the traditional method for cancer classification and grading, yet human review has limitations that can result in low reproducibility and inter-observer agreement. Computerized image analysis can partially overcome these shortcomings due to its capacity to quantitatively and reproducibly measure histologic structures on a large-scale. In this paper, we present an end-to-end image analysis and data integration pipeline for large-scale morphologic analysis of pathology images and demonstrate the ability to correlate phenotypic groups with molecular data and clinical outcomes. We demonstrate our method in the context of glioblastoma (GBM), with specific focus on the degree of the oligodendroglioma component. Over 200 million nuclei in digitized pathology slides from 117 GBMs in the Cancer Genome Atlas were quantitatively analyzed, followed by multiplatform correlation of nuclear features with molecular and clinical data. For each nucleus, a Nuclear Score (NS) was calculated based on the degree of oligodendroglioma appearance, using a regression model trained from the optimal feature set. Using the frequencies of neoplastic nuclei in low and high NS intervals, we were able to cluster patients into three well-separated disease groups that contained low, medium, or high Oligodendroglioma Component (OC). We showed that machine-based classification of GBMs with high oligodendroglioma component uncovered a set of tumors with strong associations with *PDGFRA* amplification, proneural transcriptional class, and expression of the oligodendrocyte signature genes *MBP*, *HOXD1*, *PLP1*, *MOBP* and *PDGFRA*. Quantitative morphologic features within the GBMs that correlated most strongly with oligodendrocyte gene expression were high nuclear circularity and low eccentricity. These findings highlight the potential of high throughput morphologic analysis to complement and inform human-based pathologic review.

## Introduction

Pathology images contains a wealth of phenotypic information that can be linked to underlying molecular alterations and clinical outcomes, potentially providing a high-throughput methodology for clinical diagnosis and investigation [1-6]. Modern slide scanners now produce high-resolution images within minutes, while computational and storage infrastructures have also been improved to enable high performance and parallel computation with large I/O throughput support. Furthermore, image-processing algorithms have advanced substantially to accommodate a wide range of analyses [7,8].

Microscopic features of cancer, such as tumor cell morphology, type and degree of vasculature, presence of inflammatory cells, and extent of necrosis, among others, are measurable and have biologic, diagnostic and therapeutic significance [9,10]. While human review of histologic sections has served admirably for diagnosis and research for over a century, this approach has inherent limitations for large-scale quantitative analysis and processing. Computerized image analysis could add value by scaling up and enhancing the measurement of complex micro-anatomical features [11]. Machine-based analysis also extends the scope of descriptive features beyond those readily perceived by the human visual system and offers a large number of tools for unbiased and reproducible measurements [12-15]. 

**Figure 1:**
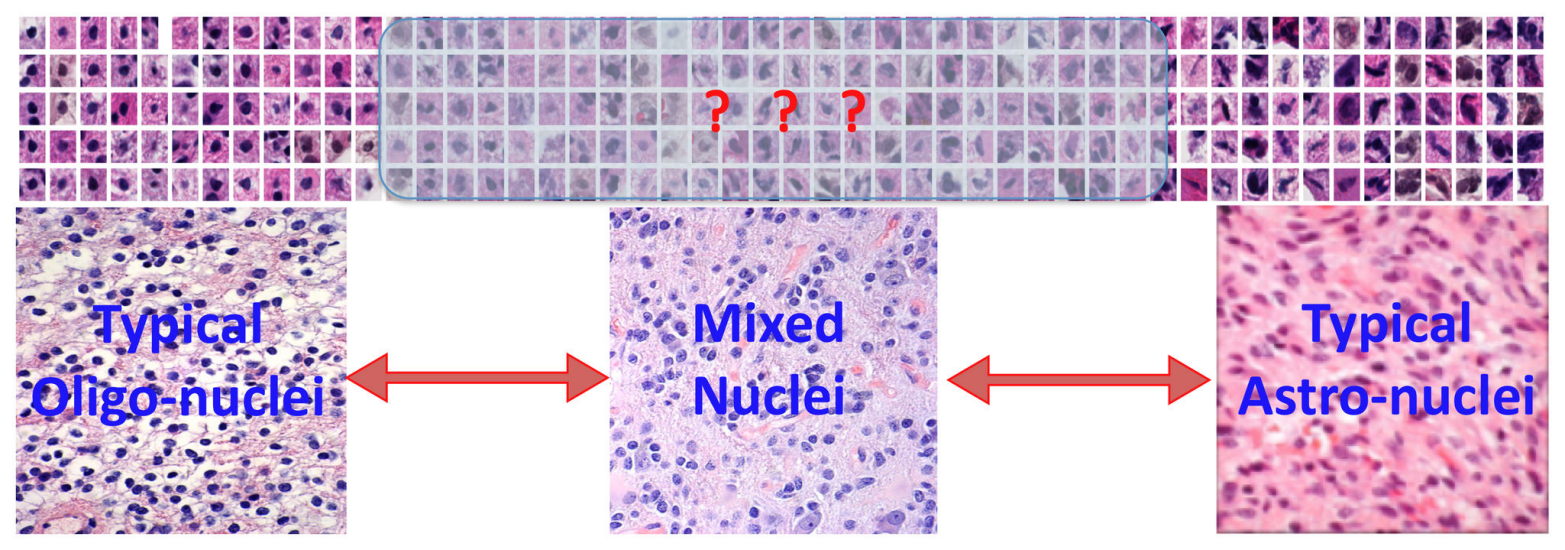
A wide morphologic spectrum of nuclei in GBMs with variable combinations of oligodendroglioma and astrocytoma features. A subset of GBMs, defined as grade IV astrocytic neoplasms, exhibits a variable degree of oligodendroglioma morphology. The shaded interval consists of a continuum of morphologies across the oligodendrolgioma to astrocytoma spectrum. Variable combinations of oligodendroglioma and astrocytoma cells, as well as morphologically ambiguous forms, make it challenging to reproducibly and accurately subclassify GBMs based on oligodendroglioma component.

Classified as the highest-grade astrocytoma, glioblastoma (GBM) has an aggressive clinical course and is fatal, generally within two years. An important subset of GBM displays variable degrees of oligodendroglioma morphology in addition to the dominant astrocytoma component [9,16-18]. Pure oligodendrogliomas tend to grow more slowly and have longer survivals, grade-for-grade, than astrocytomas. Oligodendrogliomas have morphologic characteristics that can be relied upon to distinguish them from astrocytomas, such as rounder, smaller nuclei with relatively uniform nuclear textures. By contrast, astrocytoma nuclei are elongated, irregular and have uneven nuclear textures [9,10]. In most cases, GBMs contain a heterogeneous population of neoplastic cells that span a wide morphologic spectrum consisting of numerous intermediate forms (Figure 1). The large number of tumor cell nuclei in a typical GBM (multiple millions) and the subtle differences in morphologic features make an objective and reproducible classification of these tumors challenging for neuropathologists. 

 Here we describe a new methodology for quantitative characterization of biologically meaningful morphologic components in large-scale whole slide microscopic images using a cohort of GBM samples collected by The Cancer Genome Atlas (TCGA) project [19]. We also present methods to integrate multimodal data across dimensions of clinical outcome, tumor cell morphology and molecular endpoints. In this analytic pipeline, TCGA GBMs were decoded by feature analysis of hundreds of millions of nuclei in whole slide images and clustered into cohesive groups based on the degree of Oligodendroglioma Component (OC). These machine-clustered groups were then correlated with patient outcome, transcriptional class and genetic alterations. We also compared machine-derived with human-derived patient stratification to determine if clinical or molecular correlates differed substantially. Signature genes correlated with the greatest degree of oligodendroglioma component were identified, as were specific nuclear features associated with oligodendrocyte gene expression [20,21]. Although we present our analysis framework with application to GBM, it could potentially be tailored to a broad scope of diseases since many tumor classification schemes rely on nuclear feature analysis. 

## Materials and Methods

### Ethics Statements

All data related to human subjects used for this study is de-identified and publicly available from The Cancer Genome Atlas project [19]. Therefore, this research is not classified as a human subject research and no Institutional Review Board approval is required.

### Image Dataset and Oligodendroglioma Component Annotations by TCGA Neuropathologists

Digitized microscopic images of TCGA GBM pathology slides were downloaded from the TCGA portal [19]. Digitized slides were from Hematoxylin and Eosin (H&E) stained permanent sections of tissues that were formalin-fixed and paraffin-embedded. All slides were scanned at 20X magnification. The size of the complete image data set for study is approximately 175 Gigabytes with JPEG compression ratio of 5.11.

A team of eight TCGA consortium neuropathologists annotated digitized histologic slides of TCGA cases for 18 histopathologic features. One of these 18 features was the degree of Oligodendroglioma Component (OC). The number of slides available for review ranged from 1-9 per case (median, 3). OC and all other histopathologic features were categorized as absent (0), present (1+) or abundant (2+) by two neuropathologists and adjudicated by a third.

### Image Analysis and Information Integration

**Figure 2:**
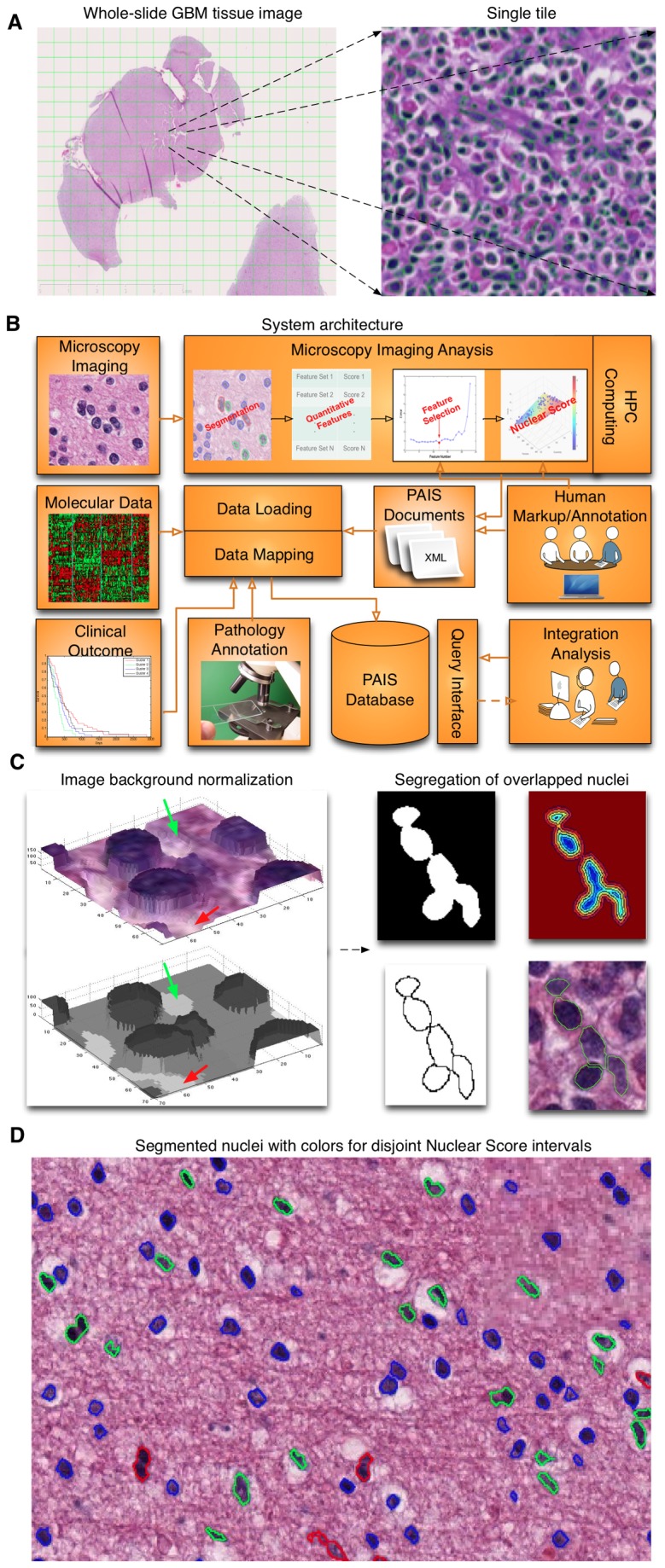
Overall schema of image analysis pipeline for GBM nuclei analysis. (A) Parallel computation mechanism. Whole-slide pathology images for analysis were partitioned into smaller image tiles for parallel processing with a computing cluster infrastructure. (B) Schematic view of the system involving data collection, annotation, analysis and integration. All data elements, including microscopy imaging features, molecular data, clinical outcomes, and expert pathology review results are stored in a centralized Pathology Analytical Imaging Standards (PAIS) database, allowing researchers to query. The module for analysis and query of histologic features consists of image analysis, parallel high performance computation, analytical result and provenance data representation, creation of interfaces for human markup and annotation acquisition, data management, query support and data sharing. (C) Nuclei segmentation method. All nuclei were segmented by an efficient segmentation method where image morphological reconstruction and the watershed algorithm were used to normalize background and to segregate clumped nuclei, respectively. (D) Visualization of nuclei with distinct Nuclear Scores (NS). An NS was calculated with a set of most discriminating features derived from the associated segmented nucleus. We use color blue, green, and red to indicate nuclei with low, median, and high NS.

**Figure 3:**
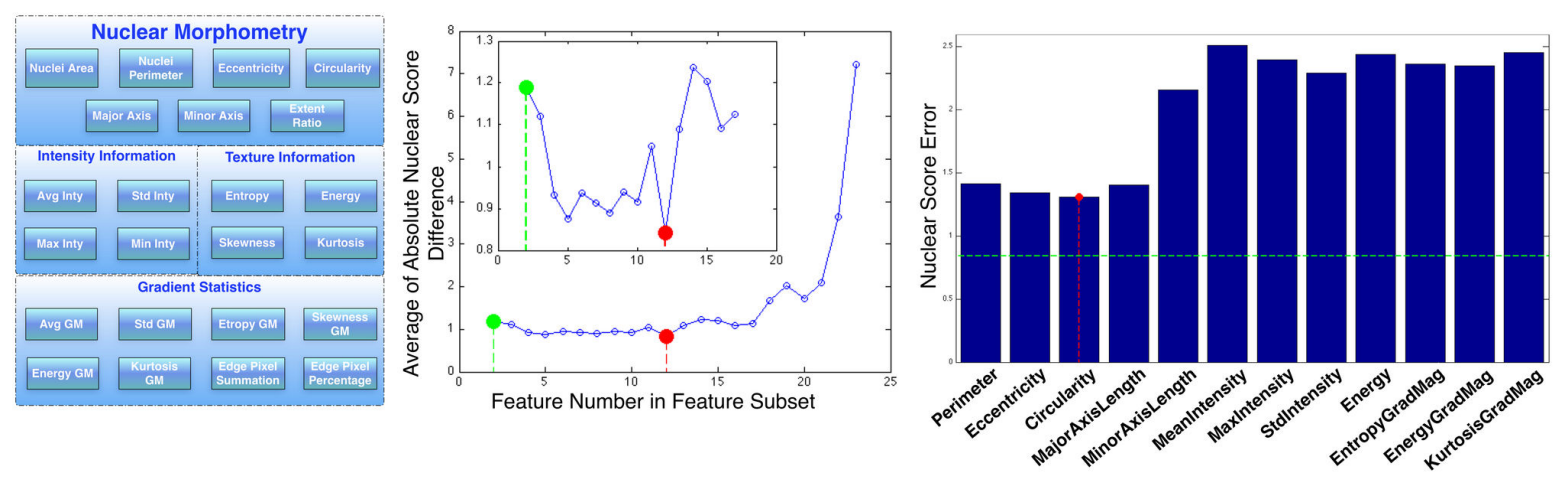
Nuclear features and discriminating feature selection. (**A**) Nuclear features can be divided into four categories: morphometry, intensity, texture, and gradient statistics. (**B**) To have the optimal representation of nuclear morphology, we plotted average absolute nuclear score difference associated with increasing numbers of selected features for the regression validation. We obtained a subset of discriminative features for Nuclear Score (NS) estimation for each given feature number, ranging from 2 to 23. The average absolute nuclear score difference reached a minimum with 12 selected features. (**C**) We also studied the histogram of average cross-validation errors associated with 12 individual features from the original feature set. Morphometry features had better performances than those of other categories. Of the morphometry features, eccentricity and circularity had the lowest NS estimation error (red dashed line). When the combined 12 features were used, the NS estimation error was lower than any single feature (green dashed line).

Whole-slide microscopic images were scanned at high resolution and often exceeded 1GB. Images were therefore partitioned into non-overlapping 4096x4096-pixel tiles to accommodate analysis requirements on a single machine and to enable parallel analysis (Figure 2A). Following the workflow illustrated in Figure 2B, we segmented approximately 200 million nuclei from 117 GBMs and represented each nucleus with a set of complementary features from four categories: morphometry, intensity, texture, and gradient statistics (Figure 3A). We obtained the optimal subset of features exhibiting the strongest discriminating power for nuclei recognition with a two-fold cross validation process (Figure 3B). We defined the Nuclear Score (NS) of a nucleus as a scalar ranging from 1 to 10, with 1 representing a classic oligodendroglioma and 10 a classic astrocytoma. Values between 1 and 10 represent nuclear features across the oligodrengroglioma-astrocytoma continuum (Figure 1). Given this definition, we applied the generalized linear regression function to NS computation with optimal nuclear representation [22]. A graphical interface was developed to facilitate collection of NS from human annotators for purposes of regression model training (Figure S1). Additionally, we developed methods for machine-based GBM stratification in terms of quantified Oligodendroglioma Component Percentage (OC%) calculated with counts of nuclei in low and high NS intervals. We selected optimal low and high NS intervals to maximize the separation power for differentiating the cases annotated by TCGA neuropathologists as low Human-graded OC (HOC) and high HOC groups. To match the human reviewing protocol, which included three categories (0, 1+ and 2+) we clustered patients into three Machine-derived OC (MOC) groups by K-means algorithm with 10000 distinct seeds for initialization. These methods allowed investigation of OC concordance between human reviewers and machine algorithms; statistical correlations with survival, treatment response, transcriptional class, genomic alterations, and histology annotations; identification of genes differentially expressed across OC groups and genes that correlated with OC%s; and revealed morphologic features most associated with oligodendrocyte signature genes. In particular, Significance Analysis of Microarrays (SAM) tests with gene expression data of patients in distinct MOC groups identified specific oligodendrocyte genes (Table 1). Further analyses of significant genes from SAM revealed the most significant annotations on their biological functions using the DAVID database [21] (http://david.abcc.ncifcrf.gov/).

**Table 1:** Summary of concordance and genomic correlates of Human-annotated (HOC) and Machine-derived Oligodendroglioma Component (MOC) groups.

**Associations with/of**	HOC-0	HOC-1	HOC-2	MOC-0	MOC-1	MOC-2
**Concordance**	Enriched by MOC-0	Similar to MOC-1	Enriched by MOC-2	Enriched in HOC-0	Similar to HOC-1	Enriched in HOC-2
**Gene Expression Class Associations**	Depleted by Proneural	Enriched by Proneural; Depleted by Mesenchymal	Enriched by Proneural	Enriched by Classical	None	Enriched by Proneural Depleted by Classical
**Somatic Mutation Associations**	Depleted by *EGFR* mutant and *PIK3R1* mutant	Enriched by *EGFR* mutant and *PIK3R1* mutant	None	Enriched by *PTEN* mutant and *RB1* mutant	Depleted by *PTEN* mutant	None
**Copy Number Variation Associations**	Depleted by amplified *PDGFRA*	None	None	None	None	Enriched by amplified *PDGFRA*
**Gene Expression Associations**	121 over expressed; 136 under expressed	None	None	25 over expressed; 242 under expressed	2 under expressed	MOBP and MBP over expressed (4 total)
**Genes Highly Correlated With OCP**	None			MBP, HOXD1, PLP1, MOBP, and PDGFRA (194 total)		

Correlation of HOC/MOC groups with transcriptional class, genomic alteration, gene expression data uncovered significant associations between OC groups and molecular data. Significance analysis of microarrays (SAM) tests identified significantly increased expression of expression of specific oligodendrocyte genes in the MOC2 group.

### Nuclei Segmentation and Feature Extraction

We focused segmentation efforts on extracting foreground objects of interest, i.e. nuclei, from background signals that displayed large variations. We used a fast hybrid grayscale reconstruction algorithm to normalize background regions degraded by artifacts introduced from the tissue preparation and scanning stage [23]. This enables the separation of foreground from normalized background with a simple threshold-based mechanism, which is key for efficiently segmenting nuclei with distinct features (Figure 2C). Overlapped nuclei were subsequently separated using the watershed technique [24]. In the post-processing step, detected objects not satisfying either area or shape constraints were filtered.

Segmentation of nuclei was followed by computation of four complementary categories of nuclear features. Visual clues used by human reviewers for discriminating oligodendroglioma from astrocytoma nuclei were primarily derived from nuclear morphology, such as size and shape. Nuclear texture features based on the chromatin content and distribution were also distinguishing among GBM subtypes. Nuclear intensity and gradient statistics, which the human eye has difficulty perceiving, were also included. For optimal nuclear representation, we retained only a subset of discriminative features for oligodendroglioma component quantification in the downstream analysis. The most discriminating feature subset consisted of features from all four categories, confirming the benefits of complementary descriptors.

### Feature Selection and Nuclear Score Computation

Indiscriminate inclusion of excessive features could result in undesirable computational cost, a known source of dimensionality challenges, and is associated with the peaking phenomenon [25]. Therefore, we investigated the discriminating power of individual features and their contribution to classification performance. To retain physical meanings of features while reducing dimensionality, we chose a subset of features with the best discriminating power in the absence of feature transformations. This is known as feature selection, an optimization process where a given set of features is reduced to a subset maximizing some user-defined objective function. We used the Sequential Floating Forward Selection (SFFS) procedure to narrow the “discriminating” features [26]. To use the SFFS algorithm, we defined the objective function as:

$$J\left(f(t)\right)=\frac{1}{N}\sum_{i=1}^{N}\left|y_{i}-f_{i}(t)\theta \left(f(t)\right)\right|$$ (1)

where *f*_*i*_(*t*) and*y*_*i*_are the selected feature vector at time t, and human-annotated NS for nucleus sample*i*, respectively; *θ*(*f*(*t*))is the trained regression function coefficient vector associated with selected feature vectors at time t for the N sample nuclei.

With the optimal selected feature subset for nuclear representation, we calculated the Nuclear Score (NS) for each nucleus to quantitatively characterize its OC degree. We used the generalized linear regression function for NS computation, because it focuses on revealing the dominant patterns between NS and nuclear features and is less subject to over-fitting than non-linear ones. However, linear least-square estimates are subject to both outliers and heavy-tailed error distribution. Therefore, we used the Iteratively Reweighted Least-Square criterion (IRLS) to mitigate the influence from outlier data [27]. Using the converged coefficients from this regression model, we calculated NS for each nucleus identified in images (Figure 2D).

To obtain the optimal feature subset, we used an independent set of five GBM images from the Emory University Hospital archives for NS regression training. Using the user interface demonstrated in Figure S1, neuropathologists labeled a set of representative nuclei covering the whole NS spectrum, ranging from 1 to 10. We ran our image analysis pipeline (i.e. segmentation and feature computation) on these images and recorded features of those nuclei. With the feature selection process completed in the cross validation paradigm, we found the optimal subset of features for nuclear representation (Table 2).

**Table 2:** Optimal feature subset for Nuclear Score (NS) discrimination.

**Feature Category**	**Feature Name**
**Geometric Shape**	Perimeter, Eccentricity, Circularity, MajorAxisLength, MinorAxisLength
**Intensity**	MeanIntensity, MaxIntensity, StdIntensity
**Statistics**	Energy
**Gradient**	EntropyGradMag, EnergyGradMag, KurtosisGradMag

Based on the average absolute Nuclear Score difference in the cross-validation process, the following 12 features were selected for computing Nuclear Score (NS). Note that the selected features are distributed across all defined feature categories, emphasizing the benefit of complementary features.

### Statistical methods

Hypergeometric testing was performed to determine enrichment and depletion of samples categorized by one platform of data in classes defined by data of another dimension [5,28]. We considered a test with p value less than 0.05 as significant. Survival analysis was performed with survival information from TCGA portal [19], where survival was interpreted as “days to death” for non right-censored patients, and “days to last follow-up” for right-censored patients. Log rank test was used to reveal significant survival difference [29,30]. Additionally, Significance Analysis of Microarrays (SAM) was employed to identify gene expression differences with data collected with Affymetrix HT-HGU133 mRNA platform [20]. SAM computes a statistic for each gene, measuring the strength of the relationship between gene expression and the response variable. It uses repeated data permutations to determine if gene expression is significantly related to the response variable.

## Results

### Assessment of Nuclear Scores and Morphology

**Figure 4:**
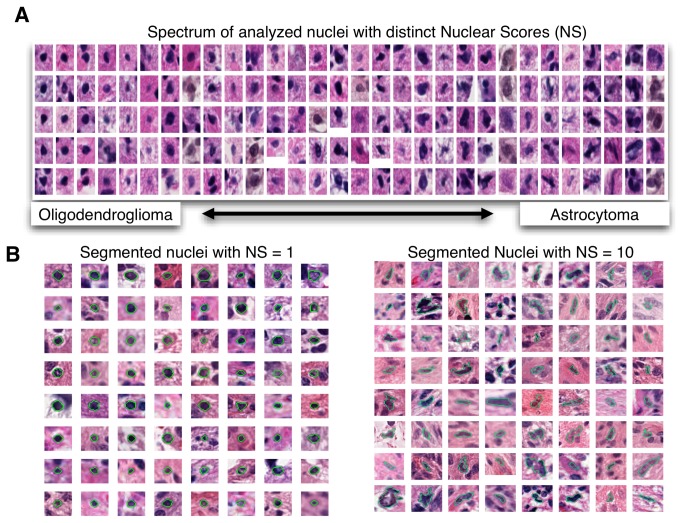
Validation of Nuclear Scores (NS) by reviewing nuclear appearances. (**A**) Spectrum of analyzed nuclei with distinct NS. Nuclei from feature-driven clusters (columns) were reviewed and the correlation between OC and NS was confirmed. (**B**) Arrays of GBM nuclei grouped by machine-classified NS. To validate results further, we aggregated 84 nuclei with (left panel) NS 1 and 84 nuclei with (right panel) NS 10, scored by machine-based regression analysis. Segmented nuclear boundaries (in green) produced by machine algorithms are overlaid. Visual and quantitative assessments verify that nuclei with NS 1 are typical of oligodendroglioma nuclei and those with NS 10 are typical of astrocytoma nuclei.

We assessed the accuracy of machine-generated Nuclear Scores (NS) by reviewing nuclei across the morphologic spectrum (Figure 4A). Nuclei were partitioned into 30 clusters by nuclear features using k-means algorithm and then sorted by the average NS for each cluster. Therefore, nuclei with each NS integer have three clusters on average to characterize the standard nuclear appearance, and morphologies of those with moderately positive and negative NS variation. Five nuclei from each cluster were randomly selected and visually reviewed. The change in nuclear morphologies matched the expected variation in the spectrum from oligodendglioma to astrocytoma according to classic neuropathology descriptions. To further validate results, we aggregated a panel of 64 randomly selected nuclei with an NS 1 and another with 64 nuclei with an NS 10 (Figure 4B). Visual assessment of machine-scored NS 1 nuclei validated them as classic for oligodendroglioma with an average NS of 1.2 by a neuropathologist’s grading (absolute machine-human NS difference: 0.23 ± 0.5). Those with an NS of 10 were classic for astrocytoma and were scored as an average of 9.7 by a neuropathologist with the absolute machine-human NS difference 0.27 ± 0.45.

### Discriminating Power of Features in Computing Nuclear Score (NS)

From the original feature set for nuclear representation (Figure 3A), we identified the optimal feature subsets, with the number of features included ranging from 2 to 23 (Figure 3B). The training error reached the minimum when the subset with 12 features was selected (Table 2). This subset included mostly morphometry features, yet all four descriptive categories were represented. To investigate the discriminant strength contributed by each selected feature, we carried out a two-fold cross-validation experiment. Recognition strength associated with morphometry features were much higher than those associated with intensity, statistics and gradient categories (Figure 3C). The only exception within the morphometry category was *minor axis length*, which had a classification error comparable to those in the other categories. *Major axis length*, on the other hand, displayed significantly less NS recognition error than *minor axis length*. Of all features in the morphometry group, *circularity* (Figure 3C) had the best discriminating performance. However, classification performance associated with any feature alone was inferior to that of all 12 selected features.

### Patient Stratification with Oligodendroglioma Component Percentage (OC%)

The degree of Oligodendroglioma Component (OC), along with other 17 pathologic criteria, was rated as absent (0), present (1+), or abundant (2+) for GBMs from TCGA by a panel of board certified neuropathologists. For comparison with this categorization, the analysis pipeline developed here computed nuclear scores (NS) for all neoplastic nuclei in 117 TCGA GBMs, typically on the order of one million nuclei per tumor. We quantified the degree of OC for each sample by calculating the Oligodendroglioma Component Percentage (OC%) using counts of nuclei within low and high NS intervals. To achieve the optimal separation power, we investigated multiple NS intervals representing oligodendroglioma and astrocytoma nuclei and various weighting functions for regression analysis. As a measure for separation power, we used GBMs categorized by TCGA neuropathologists as having low and high Oligodendroglioma Component (HOC 0 against HOC 2) and computed the p-value of the pair-wise t-test with machine-calculated OC%s. After reviewing the resulting p-values (Table S1), we selected the NS intervals and weighting function yielding the lowest p-value. We also confirmed the optimal NS intervals by testing on five sample sets, each with 80% of patients included and distinct 20% held-out. The optimal separation was noted when we included nuclear scores from 1 to 2 as our definition of oligodendrolgioma and those from 6 to 10 as our definition of astrocytoma. With these low (oligodendroglioma) and high (astrocytoma) NS intervals, the oligodendroglioma component percentage (OC%) at the patient-level was calculated as (low NS nuclei)/(low + high NS nuclei). 

**Figure 5:**
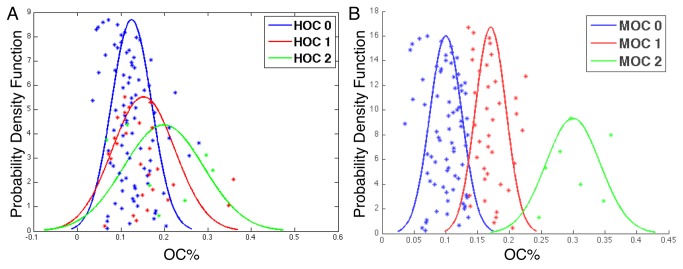
Comparisons of Oligodendroglioma Component Percentages (OC%) in Human-annotated (HOC) and Machine-derived Oligodendroglioma Component (MOC) groups. Estimated Gaussian distributions of OC%s for patients reviewed as OC 0, OC 1, and OC 2 groups by (**A**) TCGA neuropathologists; and (**B**) machine clustering approach, with NS intervals [1,2,6-10] for oligodendroglioma and astrocytoma nuclei.

We studied the resulting scatter plots and estimated Gaussian distributions of OC% associated with 117 patients from three HOC groups (Figure 5A). The pair wise t-test with OC% of the HOC 0 and those of HOC 2 patients yielded a p-value of 0.0382. Thus, the human defined groups based on oligodendroglioma component showed significant differences in OC% as determined by machine analysis. We next used an unsupervised K-means clustering algorithm with 10000 seed points to reliably partition patients into three Machine-derived OC (MOC) groups on the basis of their OC%s. These three machine-clustered groups were compared to the three patient groups determined by TCGA neuropathogists based on OC ratings [31]. The estimated Gaussian distributions of the OC% (Figure 5B) clustered by machine were well separated across MOC groups. The resulting p-value of pair wise t-test between patients of MOC 0 and those of MOC 2 was 5.98e-6. We noticed that human- and machine-based approaches stratified patients with a moderate amount of overlap, agreeing on 62% (73 out of the 117) of patients with regard to OC group assignment. Using the hypergeometric tests [5,28], we found that MOC 0 patients were enriched in HOC 0 and that patients in MOC 2 were enriched in HOC 2 (Table S2). Enrichment of MOC 1 samples in HOC 1 was just above the significance level.

### Feature Differences between Oligodendroglioma Component (OC) Groups

**Figure 6:**
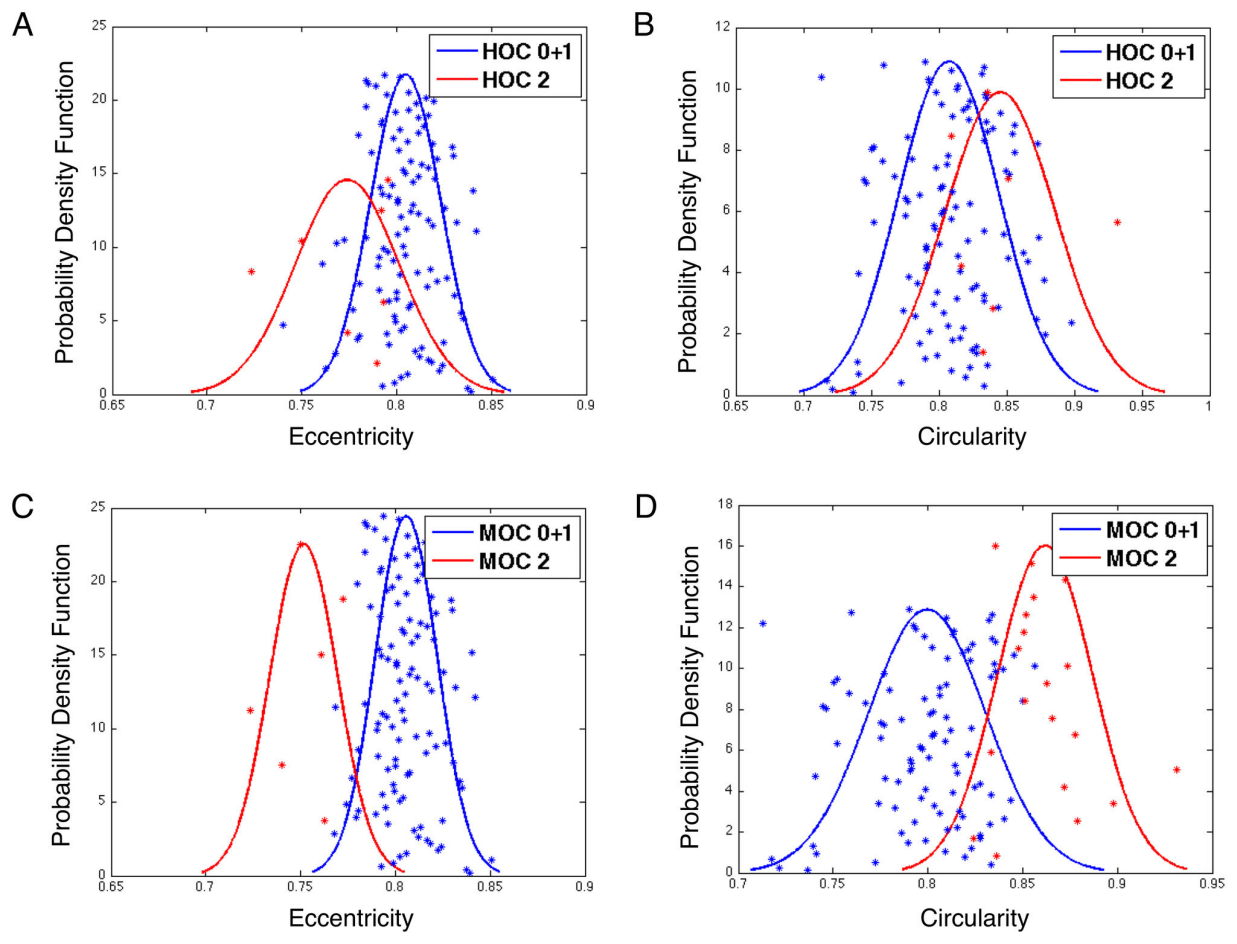
Estimated probability density functions associated with means of *eccentricity* and *circularity*. Estimated probability density functions associated with means of *eccentricity* and *circularity* demonstrate significant differences between (**A**-**B**) HOC 0+1 and HOC 2; and (**C**-**D**) MOC 0+1 and MOC 2 groups. Note that the probability density function of *eccentricity* average presents lower population mean for OC 2 group and higher mean for OC 0+1 group. Similarly, the probability density function of *circularity* average presents higher population mean in the OC 2 group and lower mean for OC 0+1 group. The findings agree with the domain knowledge of oligodendroglioma and astrocytoma nuclear morphology.

We next investigated which individual nuclear features were most discrimant between the OC groups. We calculated feature means of 12 selected features for each patient and then compared them among the OC groups with a two-sample t-test. We found that the morphologic features *eccentricity* (P = 0.02468) and *circularity* (P = 0.04819) were significantly different between HOC 0+1 and HOC 2 groups. For a determination of discriminating power of individual nuclear features, we retrained regression functions with individual selected features from the set of 12 features. At each time, we calculated a new score for each nucleus and a new OC% for each patient, followed by stratification into new MOC groups. Within these newly defined groups, we found that the feature means of *eccentricity* and *circularity* were also significantly different between patients in MOC 0+1 (i.e. MOC 0 and 1 groups in combination) and MOC 2 group (P = 5.128e-4 and P = 1.467e-10, respectively). The estimated probability density functions associated with these feature means from HOC and MOC groups are displayed in Figure 6. While taking an average operation could reduce the true signal strength substantially, we were still able to demonstrate significant separabity of *eccentricity* and *circularity* means between OC 0+1 and OC 2 groups defined by human reviewers and machine algorithm, suggesting strong correlation between these specific morphometry features and OC groups.

### Survival and Treatment Response Analysis

**Figure 7:**
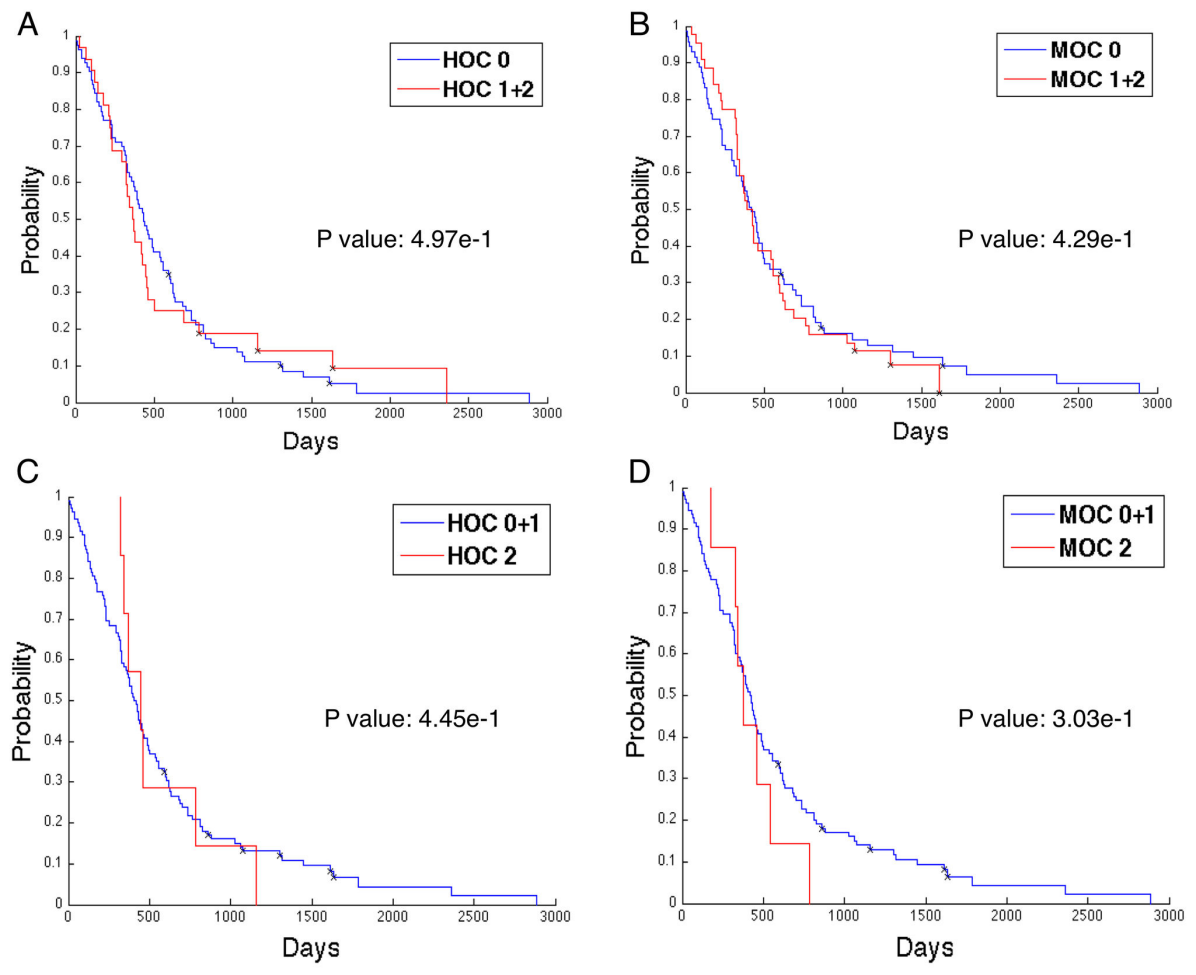
Analysis of survival with Human-annotated (HOC) and Machine-derived Oligodendroglioma Component (MOC) patient groups. Kaplan-Meier plots of the survival of TCGA GBM patients classified as OC 0 vs. those in OC 1+2 group by (**A**) TCGA neuropatholgists (P = 0.49669); (**B**) machine algorithms (P = 0.42865). Kaplan-Meier plots of the survival of TCGA GBM patients classified as OC 2 vs. those in OC 0+1 group by (**C**) TCGA neuropatholgists (P = 0.44479); (**D**) machine algorithms (P = 0.30348).

We next investigated the prognostic significance of OC designation using survival data obtained from the TCGA portal [19]. Kaplan-Meier plots [32] of patients divided by HOC and MOC groups (Figure 7) were analyzed by log rank test [29,30]. Kaplan-Meier plots for OC 0 vs. the combined group OC 1+2 stratified by human (Figure 7A) and machine (Figure 7B) did not reveal a survival difference (P = 0.49669 and 0.42865, respectively). Similarly, survivals of OC 2 vs. the combined group OC 0+1 were not significantly different for human-annotated (P = 0.44479 in Figure 7C) or for machine-derived groups (P = 0.30348 in Figure 7D). 

**Figure 8:**
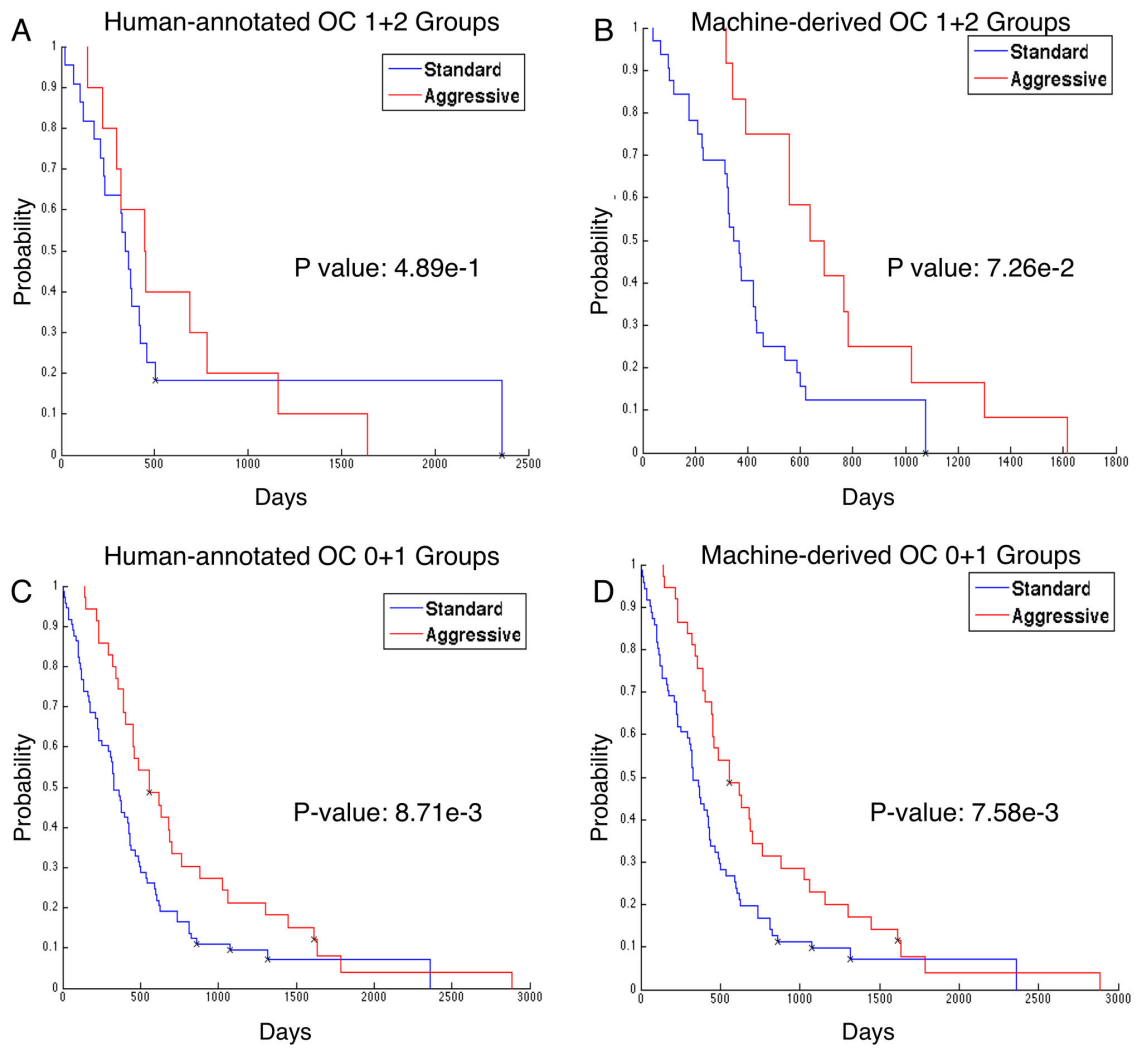
Analysis of treatment response with Human-annotated (HOC) and Machine-derived Oligodendroglioma Component (MOC) patient groups. Treatment responses of patients receiving standard and aggressive therapies are shown for patients in the OC 1+2 group defined by (**A**) TCGA neuropatholgists (P = 0.48943); (**B**) machine algorithms (P = 0.07256); and within the OC 0+1 group defined by (**C**) TCGA neuropatholgists (P = 8.71e-3); (**D**) machine algorithms (P = 7.58e-3).

We also investigated the response to therapy among and between OC groups. In the TCGA, intensive therapy was defined as three or more cycles of chemotherapy, or concurrent radiation and chemo-therapy [3,33]. Differences in outcome of patients receiving standard and intensive regimens were analyzed with the log rank test. We investigated treatment responses of patients receiving standard and aggressive therapies within the combined OC 1+2 group labeled by human reviewers (Figure 8A) and machine (Figure 8B) and identified that the response to aggressive therapy was more prominent in the MOC 1+2 than in the HOC 1+2 groups. To interrogate the significance of the observed difference in treatment effect, we performed the Cox proportional hazards regression using treatment (0 for standard and 1 for aggressive treatment) and OC group label as its predictors [34]. Based on the estimated covariate coefficient and its variance for treatment, we computed the 95% confidence interval of the hazard ratio for a one-unit change in treatment (i.e. changing from standard to aggressive treatment). The hazard ratio of aggressive treatment was 0.93 of that of the standard treatment with HOC stratification data (95% confidence interval of the hazard ratio for a one-unit change in treatment: [0.414334, 2.084136]). By contrast, the hazard ratio of aggressive treatment was 0.56 of that of standard treatment when MOC stratification data was used (95% confidence interval of the hazard ratio for a one-unit change in treatment: [0.278637, 1.105308]). While these results suggest that the treatment effect is stronger when MOC 1+2 stratification is used as compared to the HOC 1+2 stratification, the difference does not reach statistical significance. We also found significant differences in treatment response for both HOC 0+1 (P=8.71e-3, Figure 8C) and MOC 0+1 patients (P=7.58e-3, Figure 8D) with no substantial difference observed between human and machine-based stratification. In a separate comparison, we did not find any differences in survival based on treatment when we compared HOC groups to those of MOC groups. 

Transcriptional Class and Genomic Alterations Associated with Oligodendroglioma Component (OC) Groups

The TCGA has defined four clinically relevant transcriptional classes of GBMs: proneural, neural, classical and mesenchymal [33]. To investigate the potential relationship between OC groups and transcriptional classes, we performed enrichment and depletion analysis based on the hyper-geometric distribution between transcriptional classes and OC groups as defined by neuropathologists and machine algorithms [28]. We observed that proneural class cases were significantly enriched in MOC 2 (four proneural MOC 2 cases out of a total of 26 proneural and seven MOC 2 cases with P = 0.0257) and that classical class cases were enriched in MOC 0 (25 classical MOC 0 cases out of a total of 34 classical and 71 MOC 0 cases with P = 0.0480) (Table S3). In comparison, the human OC group stratification revealed an association of the proneural class with the HOC 1 and HOC 2 groups. Therefore, machine-derived patient classification revealed a specific association of proneural class with those GBMs showing the greatest degree of oligodendroglioma component. 

**Figure 9:**
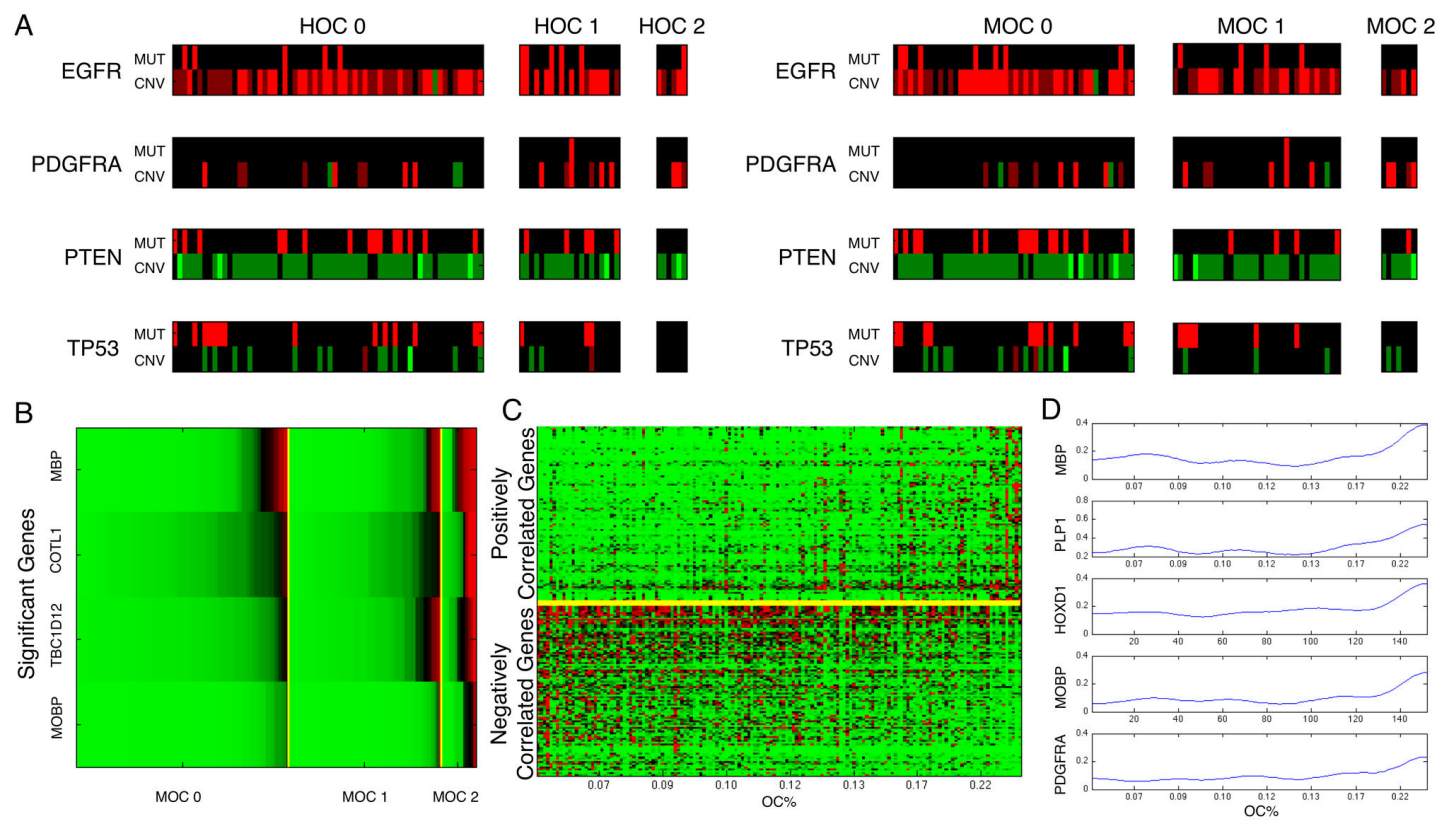
Associations of Oligodendroglioma Component (OC) groups with molecular data. (**A**) Genetic alteration profiles of GBMs in the three (left panel) HOC and (right panel) MOC groups. Mutation is depicted in red (upper row). Homozygous deletion (-2), hemizygous deletion (-1), no change (0), gain (1), and high-level amplification (2) conditions are represented in light green, dark green, black, dark red, and light red (lower row). (**B**-**D**) Analyses of MOC groups identify oligodendrocyte signature genes. (**B**) Heat map of gene expression (high expression in red) for four genes with significant overexpression in MOC 2 group compared to MOC 0+1 groups. (**C**) Heat map of expression profiles (high expression in red) of significant genes positively (upper half) and negatively (lower half) correlated with OC% by SAM. Patients are sorted on the x-axis by ascending OC%; (**D**) Smoothed expression profiles of the oligodendrocyte signature genes MBP, PLP1, HOXD1, MOBP, and PDGFRA are plotted with samples of increasing OC%.

We next studied genotype-phenotype correlations using TCGA genomic data and OC groups. For copy number variations, there are five distinct levels: homozygous deletion (-2), hemizygous deletion (-1), no change (0), gain (1), and high-level amplification (2). The overall profiles of genetic alterations associated with HOC and MOC groups are shown in Figure S2, where copy number levels of homozygous deletion, hemizygous deletion, no change, gain, and high-level amplification are represented in light green, dark green, black, dark red, and light red. Gene mutation events are depicted in red. Homozygous and hemizygous deletions were considered as a gene deletion event; only high-level amplification was considered as gene amplification event. A shorter list of hallmark genetic alterations of GBMs in HOC and MOC groups is shown in Figure 9A. We found that *PTEN* and *RB1* mutations were significantly enriched in MOC 0 groups (Table S4), with p-values of 0.0200 (15 *PTEN* mutant MOC 0 cases out of a total of 20 *PTEN* mutant and 48 MOC 0 cases) and 0.0288 (seven *RB1* mutant MOC 0 cases out of total of eight *RB1* mutant and 48 MOC 0 cases), whereas neither of these gene mutation events was significantly enriched in HOC groups (Table S5). *RB1* and *PTEN* mutations are typical of GBMs, but are found infrequent in those with an oligodendroglioma component defined by machine [35,36]. Additionally, we found a strong association between *PDGFRA* amplification and MOC 2 group (three *PDGFRA* amplified MOC 2 cases out of a total of ten *PDGFRA* amplification and six MOC 2 cases with P = 0.0131 in Table S6) whereas only a trend was noted between *PDGFRA* amplification and HOC 2 group (Table S7). *PDGFRA* amplification is tightly associated with the oligodendroglioma phenotype and data from the TCGA indicate that overexpression and/or amplification of *PDGFRA* in GBMs is typical of the proneural expression class [37,38]. 

### Pathologic Feature Associated with Oligodendroglioma Component (OC) Groups

The TCGA neuropathologists reviewed 117 GBMs based on 18 pathologic features, including necrosis, microvascular hyperplasia, inflammatory cell infiltration, tumor cell morphology, among others [19]. Each GBM was classified as absent (0), present (1), or abundant (2+) for each histopathologic feature. We investigated the associations of pathologic review features with OC groups (Table S8) and found that GBMs with no pseudopalisading necrosis were enriched in MOC 2 group. GBMs with abundant small cell morphology and sarcomatous metaplasia were enriched in MOC 0 group (Table S8), indicating that sarcomatous and small cell GBMs do not show an appreciable oligodendroglioal component [35,39]. Some common significant associations of pathologic features shared by HOC and MOC groups are listed in Table S9.

### Gene Expression Differences across Oligodendroglioma Component (OC) Groups

To identify gene expression differences across MOC groups, we applied Significance Analysis of Microarrays (SAM) [20], a statistical technique for identifying gene expression differences from data collected with Affymetrix HT-HGU133 mRNA platform. SAM computes a statistic for each gene, measuring the strength of the relationship between gene expression and the response variable (OC group). We carried out SAM analysis for each MOC group against the remaining two with a false discovery rate cutoff of < 5%. We found only four genes with significant association with MOC 2 as compared to MOC 0+1 groups. Two of them, MOBP (Myelin-associated Oligodendrocyte Basic Protein) and MBP (Myelin Basic Protein), are highly specific markers of oligodendrocytes. Sorted expression of these four genes is shown in Figure 9B. Similar significance analysis was conducted between HOC 2 and HOC 0+1 groups, yet no significant gene expression differences were found. Thus, machine-based patient stratification approach identified a group with high oligodendroglioma content that shows high expression of oligodendrocyte-specific genes. When we compared gene expression between HOC 0 against HOC 1+2 groups, we found 257 significant differences (including 121 over-expressed, and 136 under-expressed genes) (Table S10), yet no oligodendrocyte signature genes were identified. One potentially related finding was that *OLIG2* (Oligodendrocyte transcription factor 2), a universal marker of diffuse gliomas, was under expressed in HOC 0. In our analysis of MOC 0 against MOC 1+2, we found 267 significant genes (25 over expressed, and 242 under expressed) (Table S10), but no oligodendrocyte signature genes. With the same gene sets showing distinct expressions across MOC groups, we used DAVID database to further interpret gene ontology and biologic functions [21] (Table S11). 

We also carried out SAM test with the “quantitative response” to identify genes significantly correlated with the computed oligodendroglioma component percentages (OC%s). We found 194 genes (Table S10) significantly associated with the OC%s for GBMs with 5% false discovery rate cutoff (Figure 9C). Of these, five specific markers of oligodendrocytes were found: *MBP*, *HOXD1*, *PLP1, MOBP* and *PDGFRA*. The smoothed gene expression profiles of *MBP*, *HOXD1, PLP1, MOBP* and *PDGFRA* reveal increased expression with increasing OC% in GBMs (Figure 9D). Thus, the degree of oligodendroglioma component within a GBM, as defined by machine-based analysis, is highly correlated with an oligodendrocyte gene expression signature.

### Nuclear Feature Correlates of Oligodendrocyte Signature Genes

**Figure 10:**
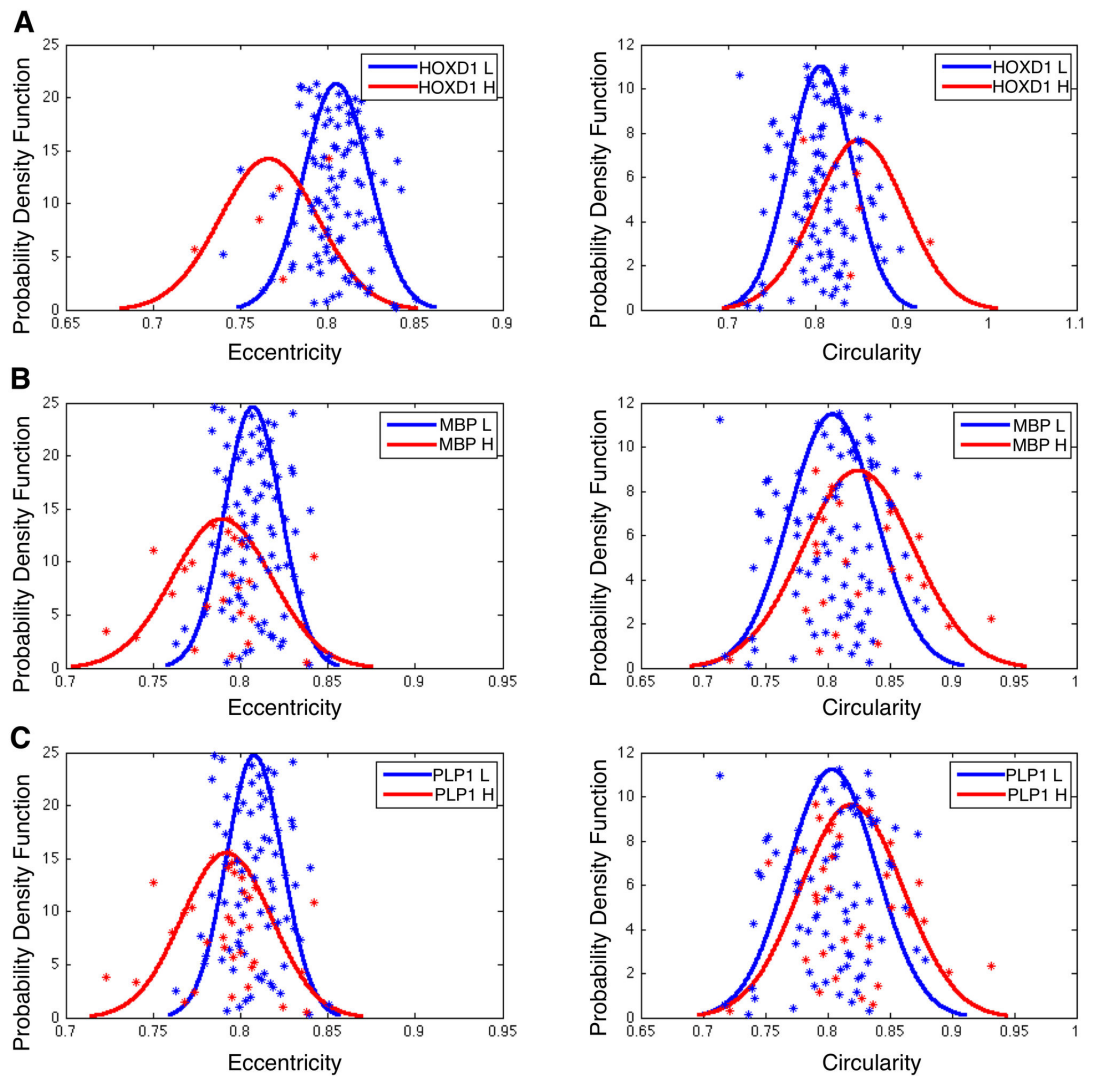
Correlation of feature means with gene expression of oligodendrocyte signature genes. Distributions associated with eccentricity and circularity means are shown for groups of low and high expression of (**A**) HOXD1, (**B**) MBP, and (**C**) PLP1, highlighting the close association between the expressions of oligodendrocyte-specific genes and nuclear features typical of oligodendroglioma.

We investigated whether specific nuclear features were correlated with the oligodendrocyte signature genes *HOXD1*, *MBP*, and *PLP1*, which were highly correlated with OC% in GBMs. We repeated the NS computation workflow used with 12 nuclear features, but with one feature at a time. For each of 12 features, we calculated its mean from all nuclei within the low and high NS intervals for each patient. For each gene of interest, we partitioned patients into low and high gene expression groups. We found that *eccentricity* means were statistically lower in the high *HOXD1* gene expression groups (P = 0.03624). *Eccentricity* was lower and *circularity* was higher in the high *MBP* expression groups (P = 7.304e-3 and P = 0.04413, respectively). *Eccentricity* was lower in the high *PLP1* expression group (P = 2.112e-3) while *circularity* trended toward being higher in this group (P = 0.06492). The resulting estimated Gaussian probability density functions associated with *eccentricity* and *circularity* means from groups of low and high expressions of gene *HOXD1*, *MBP* and *PLP1* are displayed in Figure 10. All other features means are presented in Figures S3-S5. The overall results indicate a strong link between oligodendroctye gene expression and the features of *eccentricity* and *circularity* derived from machine-based segmentation, feature extraction and NS analysis. Specifically, high oligodendrocyte gene expression is correlated with low eccentricity and high circularity. 

## Discussion

This paper presents an automated image analysis and data integration platform using whole slide pathology images, molecular data, and clinical outcome from GBM cases within The Cancer Genome Atlas. While our application focused on the degree of oligodendroglioma component within GBMs, the approach could be potentially used for other machine assisted morphologic investigations. This computational approach allowed us to quantify the morphologic composition of over 200 million nuclei in whole slide microscopic images from 117 GBMs, integrate with multi-dimension data and derive clinically meaningful molecular correlates, which is a promising approach to complement and inform human-based pathologic review.

Using machine analysis, GBMs were clustered into three well-separated groups based on their degrees of oligodendroglioma component. Our analysis indicated that machine-identified oligodendroglioma component (OC) groups were generally consistent with OC groups designated by a panel of TCGA neuropathologists [28], with the best agreement noted in low oligodendroglioma component group (OC 0). Interestingly, the morphologic features with the greatest power for discriminating oligodendroglioma component in neoplastic cells and separating patient clusters on this basis were nuclear *circularity, eccentricity, perimeter* and *major axis length*. Neuropathologists’ recognize that infiltrating glioma cells with greater circularity, less eccentricity, smaller perimeters and shorter major axis length are typical of oligodendroglioma cells and distinguish them from astrocytoma [9]. However, the ability to quantitatively assess morphologic descriptors on the scale of a million cells per tumor and to extract these four specific features as the most discriminating is beyond the capacity of human reviewers and highlights the strength of computational methods for identifying phenotypic subtypes reproducibly and accurately [13,14]. Since reproducibility and interobserver agreement in the neuropathologic classification of gliomas can be quite low, this approach and result informs human reviewers of the nuclear features that are most discriminating for recognizing oligodendroglioma within a GBM.

The advantage of using GBMs from the TCGA dataset is that morphologic features can be compared to multiplatform molecular data and clinical outcomes as ground truth. When OC groups derived from machine algorithms and human-based classifications were compared to molecular endpoints, we found that machine-based analysis could better identify tumors with an olidendroglioma molecular signature. For example, our machine-based GBM classification recognized a subset of patients with high oligodendroglioma component (MOC 2) that showed a statistically significant overexpression of genes known to be tightly associated with oligodendrocytes, including *MOBP* (encoding myelin-associated oligodendrocyte basic protein) and *MBP* (encoding myelin basic protein, the major protein of the myelin sheath). In our regression analysis, the genes that were among the most highly correlated with the degree of oligodendroglioma component percentage (OC%) were also oligodendrocyte specific, including *MBP*, *MOBP, PDGFRA, HOXD1*, a transcription factor expressed by oligodendrocytes that binds to the human myelin oligodendrocyte glycoprotein promoter, and *PLP1*, a gene specific to myelinating cells like oligodendrocytes. In contrast, the human classification of GBMs enriched by oligodendroglioma cells (HOC 2) did not reveal such an association with these oligodendrocyte signature genes. This result indicates that large-scale quantitative characterization of nuclear features by computational methods is capable of identifying a subset of GBM patients exhibiting an oligodendocyte gene signature in a manner that may complement and inform human review. 

Similarly, machine based classification of those GBMs with a high degree of oligodendroglioma component (MOC 2) revealed a statistically significant association with *PDGFRA* amplification and the proneural gene expression class, both known to be tightly correlated with the oligodendroglioma phenotype [37,38]. Furthermore, those GBMs classified with a low oligodendroglioma component (MOC 0) were strongly enriched in *PTEN* and *RB1* mutations, which is more characteristic of classic GBMs with astrocytic differentiation [35,36]. Given that these genetic correlates noted with machine-based stratification were not as evident on human-based classifications, neuropathologists could potentially use this approach as a large-scale screening or verification process.

With the ability to quantitatively analyze neoplasms at a scale not accommodated by human reviewers, machine-based methods have the potential to further inform human reviewers of discriminating features related to gene expression or genetic correlates [15]. Specific morphologic features in digitized images that are associated with gene expression patterns or clinical outcomes provide an evidence-based and clinically meaningful mechanism to subdivide diseases phenotypically. In the case of GBMs within the TCGA dataset, we demonstrated that the nuclear features that correlated most strongly with the expression of oligodendrocyte specific genes (*HOXD1*, *MBP*, and *PLP1*) were high *circularity* and low *eccentricity*. Since both oligodendrocytes and oligodendroglioma cells are characterized by round, symmetric nuclei, this result is intuitive. Moreover, this approach can be used to determine which morphologic features correspond with specific genes, genetic alterations, and signaling networks in a broader scope. Thus, our platform has the potential to inform pathologists of those these specific morphologic features that will most accurately identify a tumor element with a given gene signature.

The performance advantage of machine-based OC classification to that of traditional human-based approach, as measured by identification of significant associations with molecular endpoints, is likely due to its consistent performance on the large-scale that is not feasible for humans [11]. Since the size of the training samples (five archived images from Emory University Hospital) in our study was nominal as compared to the total number of neoplastic nuclei in TCGA whole-slide images, human-based analysis on the small set of nuclei would be expected to yield high accuracy. However, performance drastically drops when the scope of analysis is expanded to the order of millions of nuclei per tumor sample. Unlike neuropathologists, computer-based analysis is not vulnerable to analytic scale. In our study, the capacity of large-scale analysis by machines enables objective and quantitative OC measurement and the resulting discovery of significant associations that would be identified at a much larger cost of human labor and time. Moreover, machine-based analysis on digital slides extends the scope of descriptive features from those appreciated by pathology domain experts to those not perceived by the human visual detection system. In addition to the nuclear morphologic features that we found the most discriminating, we uncovered other complementary features from intensity, statistics and gradient feature categories (Table 2) that enhanced the accuracy of the nuclear score (NS) computation for oligodendroglioma component (OC) quantization. 

Although our work reveals the potential to improve and inform human disease classification, several limitations will have to be addressed before the method can have full impact on translational science. Firstly, we focused on only one attribute of GBM morphology rather than its full morphologic complexity. Features other than tumor cell morphology, such as angiogenesis, necrosis, thrombosis, lymphocytic and macrophage infiltrates are also critical to tumor behavior and will have to be considered for a comprehensive analysis [35,40]. Secondly, there is a degree of circular logic to our approach. We have asked neuropathologists to annotate a teaching set for their impression of degree of oligodendroglioma differentiation in each nucleus in order to build our regression. Thus, a subjective human-based definition of nuclear morphology was the ground truth of our analysis. As such, the findings of well-known correlations of nuclear morphology (e.g. *perimeter*, *circularity* and *eccentricity*, etc) with degree of oligodendroglial morphology or oligodendrocyte gene expression should not be unexpected. However, due to our unbiased, large scale and quantitative approach, our machine-based analysis was able to uncover molecular signatures that were not apparent following human disease classification, highlighting the strengths of computational approaches to digital pathology. Lastly, in our integrative analysis, we have focused almost exclusively on pairwise correlations rather than simultaneous analyses over multiple dimensions. In this manner, we may have missed interesting associations that can only be revealed by synergetic analyses with multi-dimensional data. This problem will be further investigated in future research. 

Overall, the analysis framework presented provides a generic approach for large-scale microscopy images and for comprehensive correlative investigations using complementary disease data. Although this study focused on the analysis of an oligodendroglioma component in GBM, the platform could potentially be generalized to other morphology-based analyses that integrate clinical and molecular data. 

## Supporting Information

Figure S1 **A graphical user interface developed to facilitate the Nuclear Score (NS) collection and training process.** To train the regression model for generating desired NS for segmented nuclei, we collected a separate set of training samples graded by human annotators with a user-friendly graphical user interface. In this process, all nuclei were pre-segmented and the associated features were pre-calculated by computer algorithms. Users can click on the nucleus of interest and efficiently choose the corresponding NS in the feature Table. After completing the training process, results were exported and saved in files on the local disk.
(TIFF)

Figure S2 **Genetic alteration profiles for TCGA GBM patients, including mutations and copy number variations.** Genetic alteration profiles are shown for TCGA patients in (**A**) HOC, and (**B**) MOC groups. Mutations are depicted in red (upper row). Homozygous deletion (-2), hemizygous deletion (-1), no change (0), gain (1), and high-level amplification (2) are represented in light green, dark green, black, dark red, and light red (lower row).
(TIFF)

Figure S3 **Estimated Gaussian probability density functions associated with means of 12 individual selected features from low and high HOXD1 gene expression groups.**
(TIFF)

Figure S4 **Estimated Gaussian probability density functions associated with means of 12 individual selected features from low and high MBP gene expression groups.**
(TIFF)

Figure S5 **Estimated Gaussian probability density functions associated with means of 12 individual selected features from low and high PLP1 gene expression groups.**
(TIFF)

Table S1 **Optimal weight function and Nuclear Score (NS) intervals for defining oligodendroglioma and astrocytoma nuclei in GBM digitized images.** P-values were computed for pair wise t-tests with OC%s of the HOC 0 patient population compared to those of HOC 2 rated by TCGA neuropathologists. Multiple definitions for NS intervals for oligodendroglioma and astrocytoma nuclei and weighting functions for regression analysis were investigated.
(DOC)

Table S2 **Concordance between Human-annotated (HOC) and Machine-derived Oligodendroglioma Component (MOC) groups.** P-values for (left) enrichment and (right) depletion analysis of MOC groups within the three HOC groups were calculated using the right and left hypergeometric tails, respectively.
(DOC)

Table S3 **Association between Human-annotated (HOC) / Machine-derived Oligodendroglioma Component (MOC) groups and TCGA transcriptional subtypes.** P-values for (left) enrichment and (right) depletion analysis of Verhaak transcriptional subtypes within HOC and MOC groups were calculated using the right and left hypergeometric tails respectively.
(DOC)

Table S4 **Associations between Machine-derived Oligodendroglioma Component (MOC) groups and gene mutations.** P-values for (left) enrichment and (right) depletion analysis of mutated genes of interest within the three MOC groups were calculated using the right and left hypergeometric tails respectively.
(DOC)

Table S5 **Associations between Human-annotated Oligodendroglioma Component (HOC) groups and gene mutations.** P-values for (left) enrichment and (right) depletion analysis of mutated genes within the three HOC groups were calculated using the right and left hypergeometric tails respectively.
(DOC)

Table S6 **Associations between Machine-derived Oligodendroglioma Component (MOC) groups and gene copy number variations.** P-values for (top row) enrichment, and (bottom row) depletion analysis of (left) genetic deletion (-2=homozygous deletion; -1=hemizygous deletion), (middle) no change (0=neutral/no change) and (right) amplification (2=high level amplification) within the three MOC groups were calculated using the right and left hypergeometric tails respectively.
(DOC)

Table S7 **Associations between Human-annotated Oligodendroglioma Component (HOC) groups and copy number variations.** P-values for (top row) enrichment, and (bottom row) depletion analysis of (left) genetic deletion (-2=homozygous deletion; -1=hemizygous deletion), (middle) no change (0=neutral/no change) and (right) amplification (2=high level amplification) within the three HOC groups were calculated using the right and left hypergeometric tails respectively.
(DOC)

Table S8 **Associations between Machine-derived Oligodendroglioma Component (MOC) groups and human-annotated histology groups.** P-values for (top row) enrichment, and (bottom row) depletion of pathologic ratings (left: absence, middle: presence, and right: abundance) within the three MOC groups were calculated using the right and left hypergeometric tails respectively.
(DOC)

Table S9 **Associations of pathologic features shared by Human-annotated (HOC) and Machine-derived Oligodendroglioma Component (MOC) groups.** We list all significant enrichment/depletion findings shared in HOC- and MOC-based correlative studies with pathologic ratings. Pathologic ratings 0, 1, 2 represent “absence”, “presence”, and “abundance”.
(DOC)

Table S10 **Full list of genes presenting 1) significance in pairwise SAM tests with Human-annotated (HOC) and Machine-derived Oligodendroglioma Component (MOC) groups; and 2) associations with Oligodendroglioma Component Percentages (OC%s) using SAM quantitative response regression.**
(XLS)

Table S11 **Gene ontology and biologic functions interpreted by DAVID databases.**
(XLS)

Text S1 **Endnote Library S1.**
(DOC)
