# Supplementary figures and images for: Machine-Based Morphologic Analysis of Glioblastoma Using Whole-Slide Pathology Images Uncovers Clinically Relevant Molecular Correlates
Source: PLoS One. 2013 Nov 13;8(11):e81049. doi: 10.1371/journal.pone.0081049 (PMC3827469; doi:10.1371/journal.pone.0081049)

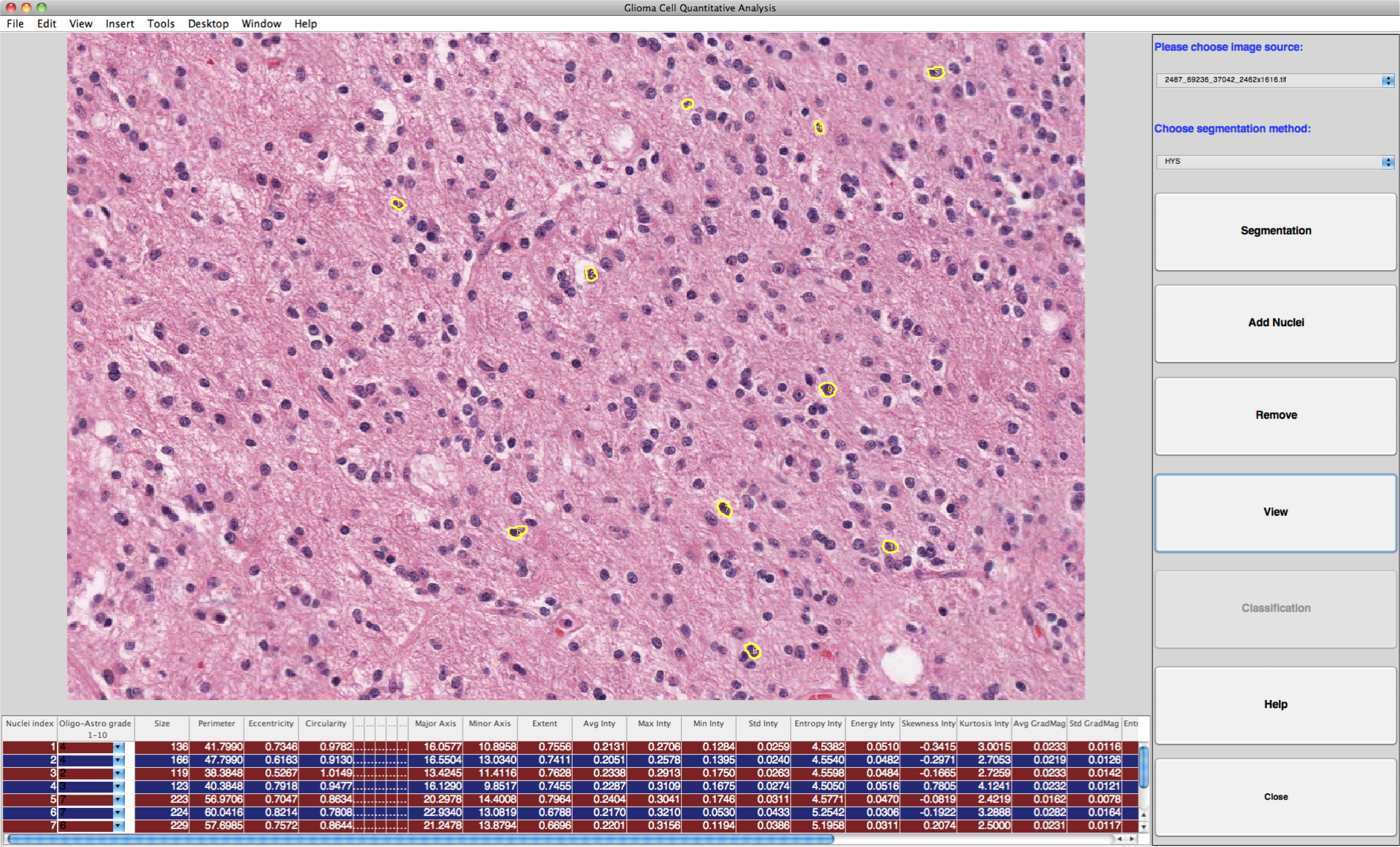

Supplement: Figure S1 — A graphical user interface developed to facilitate the Nuclear Score (NS) collection and training process. To train the regression model for generating desired NS for segmented nuclei, we collected a separate set of training samples graded by human annotators with a user-friendly graphical user interface. In this process, all nuclei were pre-segmented and the associated features were pre-calculated by computer algorithms. Users can click on the nucleus of interest and efficiently choose the corresponding NS in the feature Table. After completing the training process, results were exported and saved in files on the local disk. (TIFF) [file pone.0081049.s001.tiff]

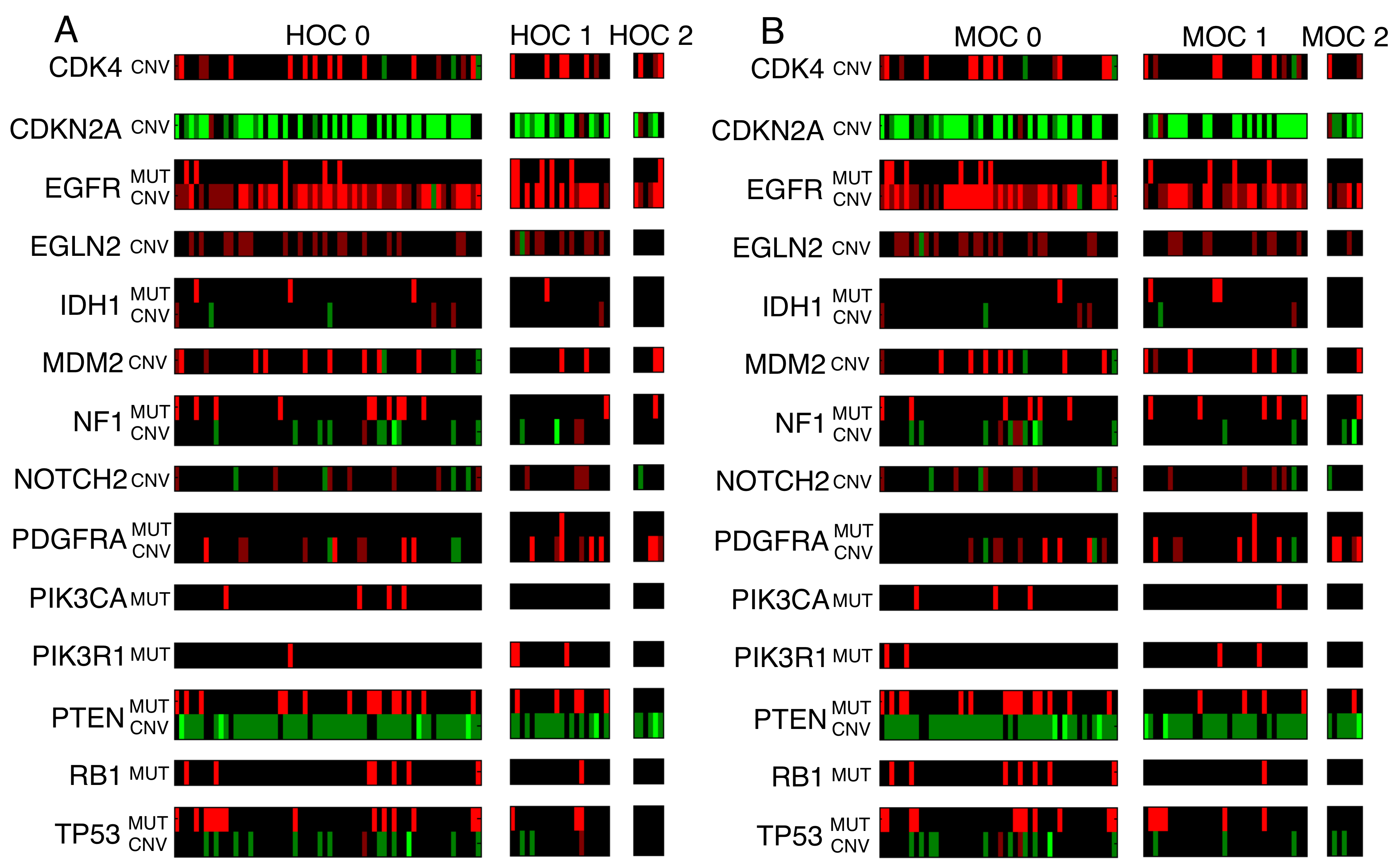

Supplement: Figure S2 — Genetic alteration profiles for TCGA GBM patients, including mutations and copy number variations. Genetic alteration profiles are shown for TCGA patients in (A) HOC, and (B) MOC groups. Mutations are depicted in red (upper row). Homozygous deletion (-2), hemizygous deletion (-1), no change (0), gain (1), and high-level amplification (2) are represented in light green, dark green, black, dark red, and light red (lower row). (TIFF) [file pone.0081049.s002.tiff]

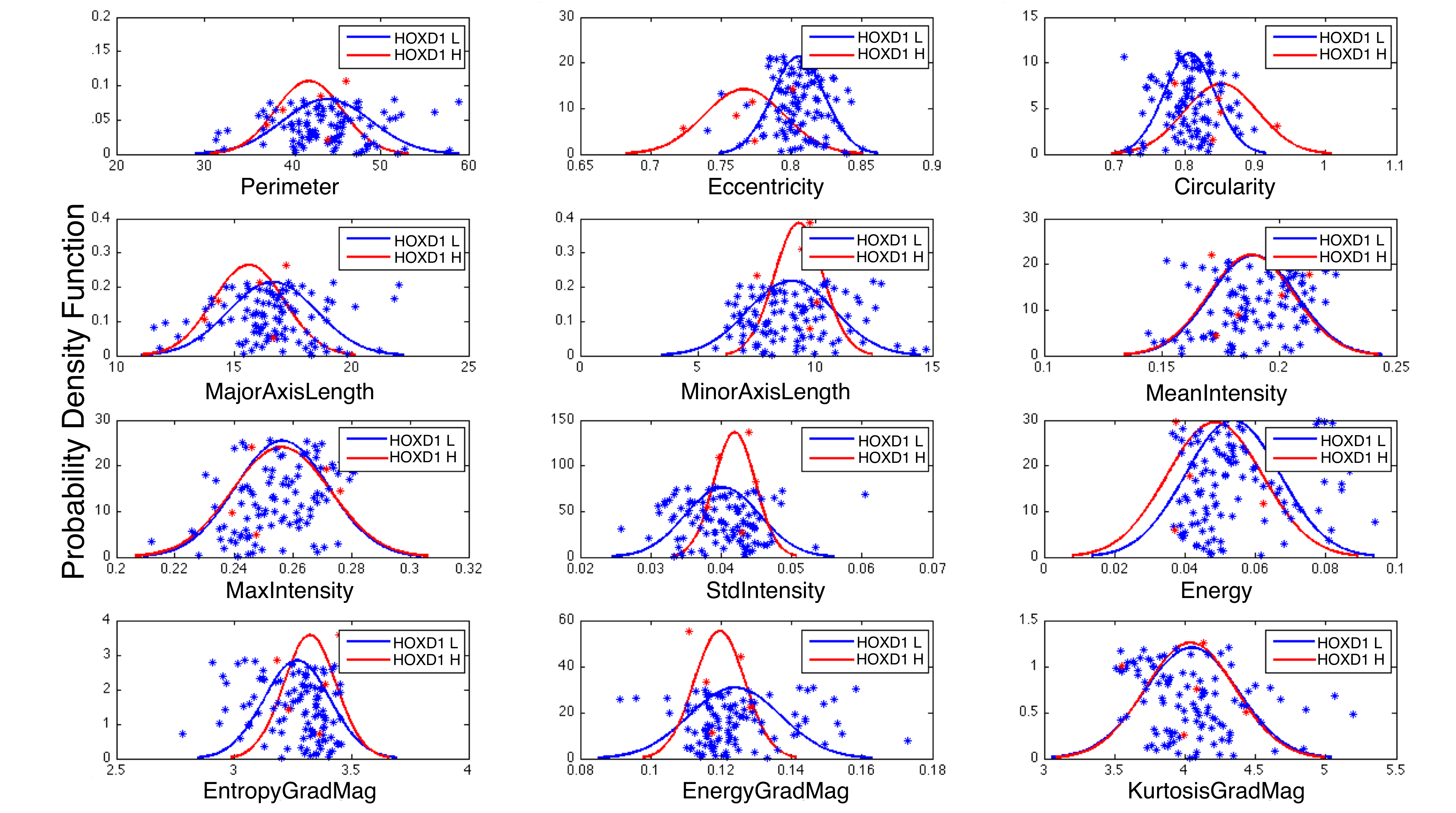

Supplement: Figure S3 — Estimated Gaussian probability density functions associated with means of 12 individual selected features from low and high HOXD1 gene expression groups. (TIFF) [file pone.0081049.s003.tiff]

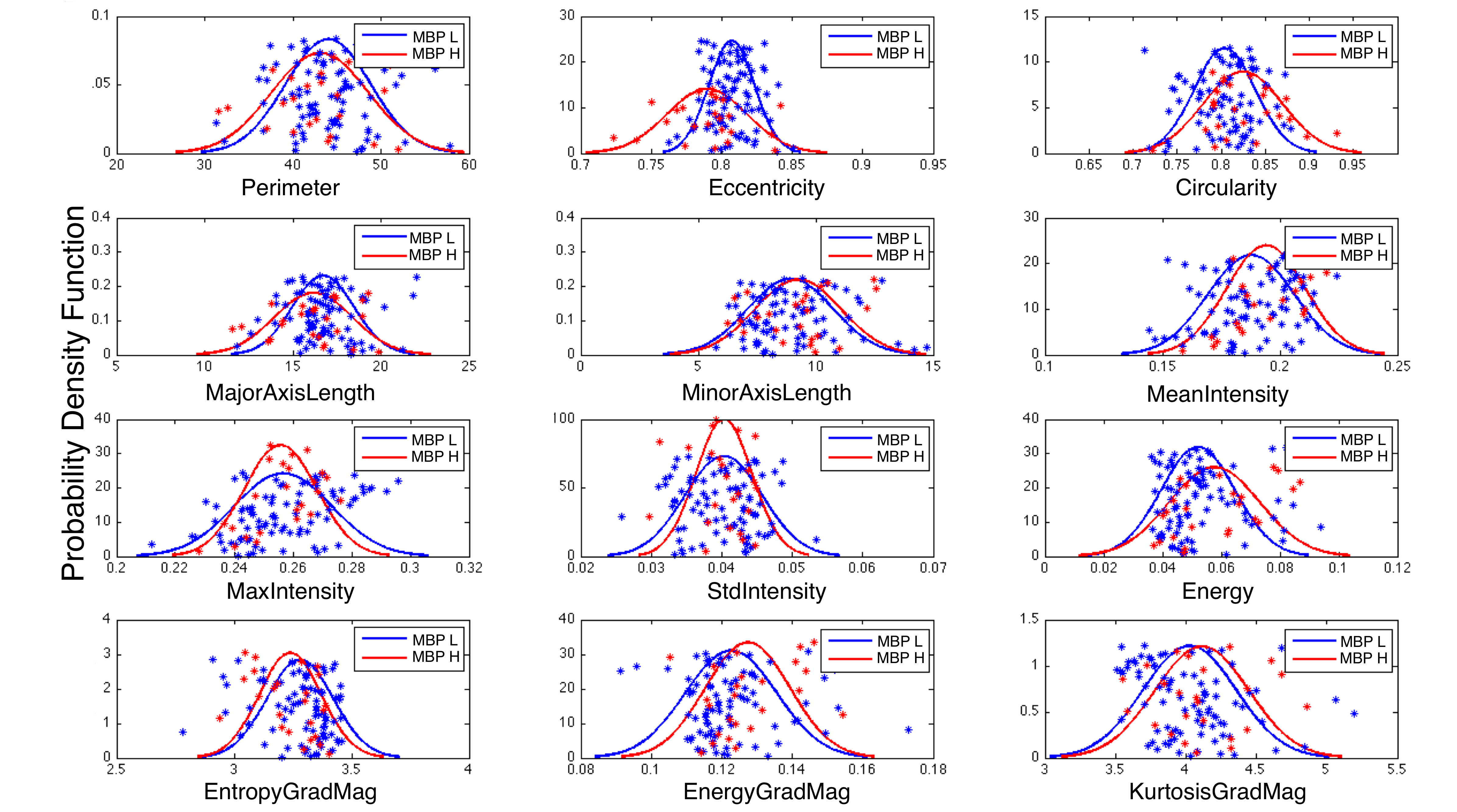

Supplement: Figure S4 — Estimated Gaussian probability density functions associated with means of 12 individual selected features from low and high MBP gene expression groups. (TIFF) [file pone.0081049.s004.tiff]

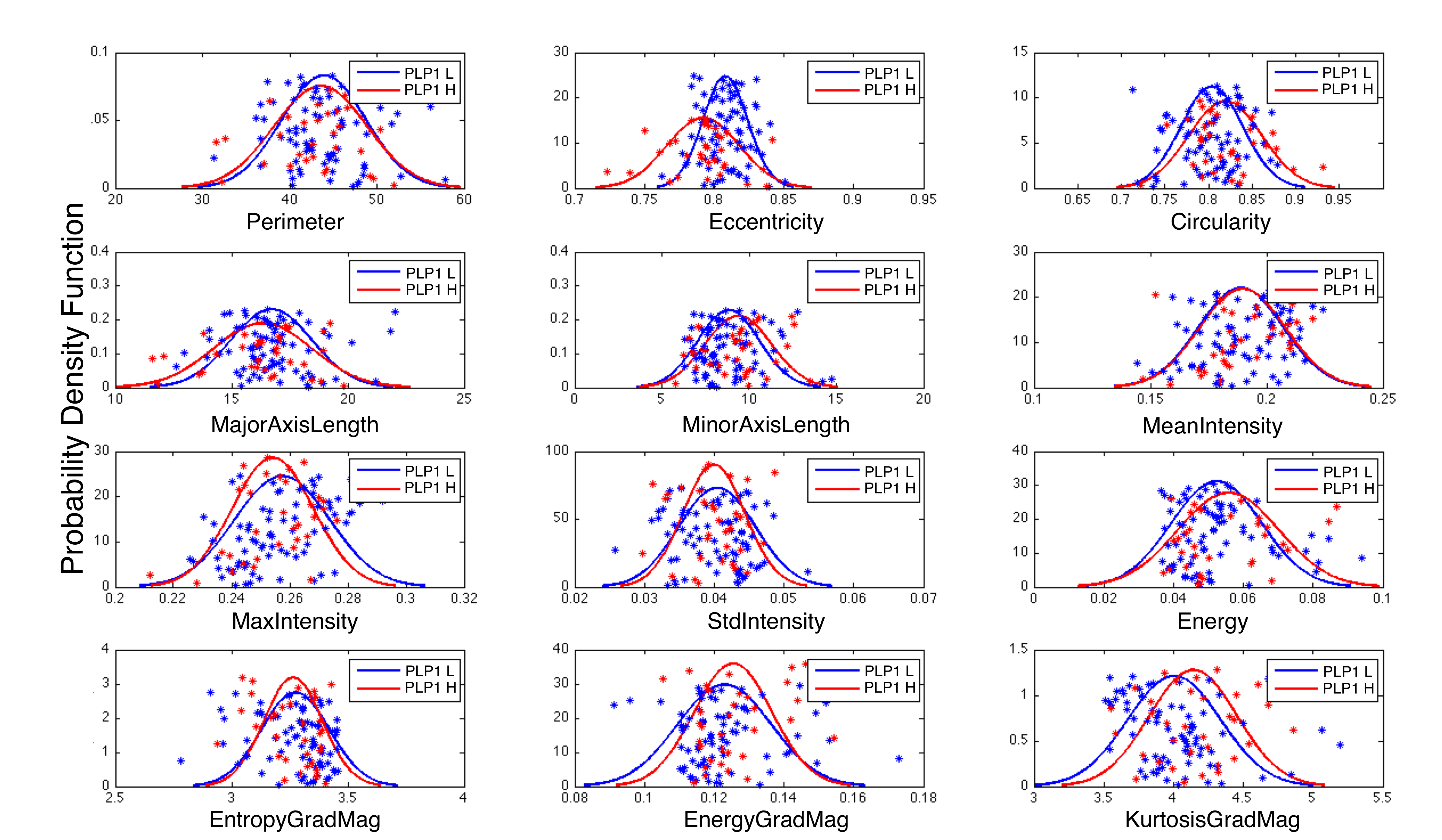

Supplement: Figure S5 — Estimated Gaussian probability density functions associated with means of 12 individual selected features from low and high PLP1 gene expression groups. (TIFF) [file pone.0081049.s005.tiff]
