# Supplementary material for: Machine-Based Morphologic Analysis of Glioblastoma Using Whole-Slide Pathology Images Uncovers Clinically Relevant Molecular Correlates
Source: PLoS One. 2013 Nov 13;8(11):e81049. doi: 10.1371/journal.pone.0081049 (PMC3827469; doi:10.1371/journal.pone.0081049)
Supplement: Table S1 — Optimal weight function and Nuclear Score (NS) intervals for defining oligodendroglioma and astrocytoma nuclei in GBM digitized images. P-values were computed for pair wise t-tests with OC%s of the HOC 0 patient population compared to those of HOC 2 rated by TCGA neuropathologists. Multiple definitions for NS intervals for oligodendroglioma and astrocytoma nuclei and weighting functions for regression analysis were investigated. (DOC) [file pone.0081049.s006.doc]

**Table S1.** Optimal weight function and Nuclear Score (NS) intervals for defining oligodendroglioma and astrocytoma nuclei in GBM digitized images. P-values were computed for pair wise t-tests with OC%s of the HOC 0 patient population compared to those of HOC 2 rated by TCGA neuropathologists. Multiple definitions for NS intervals for oligodendroglioma and astrocytoma nuclei and weighting functions for regression analysis were investigated.

| **Oligo/Astro Nuclear Score Partition** | **Weighting Functions** | | | | | | | |
| --- | --- | --- | --- | --- | --- | --- | --- | --- |
|  | Bisqure | Andrew | Cauchy | Huber | Logistic | Ols | Talwar | Welsch |
| **[1, 2] / [6, 10]** | **0.0382** | 0.0383 | 0.0400 | 0.0383 | 0.0407 | 0.0445 | 0.0445 | 0.0387 |
| **[1, 2] / [5, 10]** | **0.0414** | 0.0415 | 0.0431 | 0.0410 | 0.0437 | 0.0471 | 0.0471 | 0.0420 |
