# Supplementary material for: Machine-Based Morphologic Analysis of Glioblastoma Using Whole-Slide Pathology Images Uncovers Clinically Relevant Molecular Correlates
Source: PLoS One. 2013 Nov 13;8(11):e81049. doi: 10.1371/journal.pone.0081049 (PMC3827469; doi:10.1371/journal.pone.0081049)
Supplement: Table S2 — Concordance between Human-annotated (HOC) and Machine-derived Oligodendroglioma Component (MOC) groups. P-values for (left) enrichment and (right) depletion analysis of MOC groups within the three HOC groups were calculated using the right and left hypergeometric tails, respectively. (DOC) [file pone.0081049.s007.doc]

**Table S2.** Concordance between Human-annotated (HOC) and Machine-derived Oligodendroglioma Component (MOC) groups. P-values for (left) enrichment and (right) depletion analysis of MOC groups within the three HOC groups were calculated using the right and left hypergeometric tails, respectively.

|  | **MOC 0** | **MOC 1** | **MOC 2** |
| --- | --- | --- | --- |
| **HOC 0** | **0.0048,** 0.9952 | 0.9191, 0.0809 | 0.9897, **0.0103** |
| **HOC 1** | 0.9617, **0.0383** | **0.0520,** 0.9480 | 0.3350, 0.6650 |
| **HOC 2** | 0.9558, **0.0442** | 0.5688, 0.4312 | **0.0022,** 0.9978 |
