# Supplementary material for: Machine-Based Morphologic Analysis of Glioblastoma Using Whole-Slide Pathology Images Uncovers Clinically Relevant Molecular Correlates
Source: PLoS One. 2013 Nov 13;8(11):e81049. doi: 10.1371/journal.pone.0081049 (PMC3827469; doi:10.1371/journal.pone.0081049)
Supplement: Table S3 — Association between Human-annotated (HOC) / Machine-derived Oligodendroglioma Component (MOC) groups and TCGA transcriptional subtypes. P-values for (left) enrichment and (right) depletion analysis of Verhaak transcriptional subtypes within HOC and MOC groups were calculated using the right and left hypergeometric tails respectively. (DOC) [file pone.0081049.s008.doc]

**Table S3** Association between Human-annotated (HOC) / Machine-derived Oligodendroglioma Component (MOC) groups and TCGA transcriptional subtypes. P-values for (left) enrichment and (right) depletion analysis of Verhaak transcriptional subtypes within HOC and MOC groups were calculated using the right and left hypergeometric tails respectively.

|  | **CLASSICAL** | **MESENCHYMAL** | **NEURAL** | **PRONEURAL** |
| --- | --- | --- | --- | --- |
| **HOC 0** | 0.2608, 0.7392 | **0.0165**, 0.9835 | 0.6331, 0.3669 | 0.9966, **0.0034** |
| **HOC 1** | 0.5676, 0.4324 | 0.9501, **0.0499** | 0.3603, 0.6397 | **0.0436**, 0.9564 |
| **HOC 2** | 0.7947, 0.2053 | 0.8528, 0.1472 | 0.4556, 0.5444 | **0.0257**, 0.9743 |
| **MOC 0** | **0.0480**, 0.9520 | 0.6670, 0.3330 | 0.4799, 0.5201 | 0.9129, 0.0871 |
| **MOC 1** | 0.7952, 0.2048 | 0.1559, 0.8441 | 0.7324, 0.2676 | 0.3793, 0.6207 |
| **MOC 2** | 0.9603, **0.0397** | 0.8528, 0.1472 | 0.1531, 0.8469 | **0.0257**, 0.9743 |
