# Supplementary material for: Machine-Based Morphologic Analysis of Glioblastoma Using Whole-Slide Pathology Images Uncovers Clinically Relevant Molecular Correlates
Source: PLoS One. 2013 Nov 13;8(11):e81049. doi: 10.1371/journal.pone.0081049 (PMC3827469; doi:10.1371/journal.pone.0081049)
Supplement: Table S4 — Associations between Machine-derived Oligodendroglioma Component (MOC) groups and gene mutations. P-values for (left) enrichment and (right) depletion analysis of mutated genes of interest within the three MOC groups were calculated using the right and left hypergeometric tails respectively. (DOC) [file pone.0081049.s009.doc]

**Table S4.** Associations between Machine-derived Oligodendroglioma Component (MOC) groups and gene mutations. P-values for (left) enrichment and (right) depletion analysis of mutated genes of interest within the three MOC groups were calculated using the right and left hypergeometric tails respectively.

|  | **MOC 0** | **MOC 1** | **MOC 2** |
| --- | --- | --- | --- |
| **EGFR** | 0.2714, 0.7286 | 0.5239, 0.4761 | 0.8106, 0.1894 |
| **IDH1** | 0.8591, 0.1409 | 0.0819, 0.9181 | 0.6433, 0.3567 |
| **NF1** | 0.6288, 0.3712 | 0.3747, 0.6253 | 0.4490, 0.5510 |
| **PDGFRA** | 0.7727, 0.2273 | 0.1875, 0.8125 | 0.5398, 0.4602 |
| **PIK3CA** | 0.2318, 0.7682 | 0.6681, 0.3319 | 0.6433, 0.3567 |
| **PIK3R1** | 0.5688, 0.4312 | 0.3144, 0.6856 | 0.6433, 0.3567 |
| **PTEN** | **0.0200**, 0.9800 | 0.9653, **0.0347** | 0.6749, 0.3251 |
| **RB1** | **0.0288**, 0.9712 | 0.9290, 0.0710 | 0.7498, 0.2502 |
| **TP53** | 0.1836, 0.8164 | 0.5746, 0.4254 | 0.8953, 0.1047 |
