# Supplementary material for: Machine-Based Morphologic Analysis of Glioblastoma Using Whole-Slide Pathology Images Uncovers Clinically Relevant Molecular Correlates
Source: PLoS One. 2013 Nov 13;8(11):e81049. doi: 10.1371/journal.pone.0081049 (PMC3827469; doi:10.1371/journal.pone.0081049)
Supplement: Table S5 — Associations between Human-annotated Oligodendroglioma Component (HOC) groups and gene mutations. P-values for (left) enrichment and (right) depletion analysis of mutated genes within the three HOC groups were calculated using the right and left hypergeometric tails respectively. (DOC) [file pone.0081049.s010.doc]

**Table S5.** Associations between Human-annotated Oligodendroglioma Component (HOC) groups and gene mutations. P-values for (left) enrichment and (right) depletion analysis of mutated genes within the three HOC groups were calculated using the right and left hypergeometric tails respectively.

|  | **HOC 0** | **HOC 1** | **HOC 2** |
| --- | --- | --- | --- |
| **EGFR** | 0.9632, **0.0368** | **0.0413**, 0.9587 | 0.3620, 0.6380 |
| **IDH1** | 0.4501, 0.5499 | 0.4358, 0.5642 | 0.6250, 0.3750 |
| **NF1** | 0.1599, 0.8401 | 0.8899, 0.1101 | 0.3920, 0.6080 |
| **PDGFRA** | 0.8523, 0.1477 | 0.1136, 0.8864 | 0.5341, 0.4659 |
| **PIK3CA** | 0.1196, 0.8804 | 0.8254, 0.1746 | 0.6250, 0.3750 |
| **PIK3R1** | 0.9590, **0.0410** | **0.0187**, 0.9813 | 0.6250, 0.3750 |
| **PTEN** | 0.3185, 0.6815 | 0.3866, 0.6134 | 0.8990, 0.1010 |
| **RB1** | 0.1521, 0.8479 | 0.7342, 0.2658 | 0.7228, 0.2772 |
| **TP53** | 0.1238, 0.8762 | 0.6927, 0.3073 | 0.8679, 0.1321 |
