# Supplementary material for: Machine-Based Morphologic Analysis of Glioblastoma Using Whole-Slide Pathology Images Uncovers Clinically Relevant Molecular Correlates
Source: PLoS One. 2013 Nov 13;8(11):e81049. doi: 10.1371/journal.pone.0081049 (PMC3827469; doi:10.1371/journal.pone.0081049)
Supplement: Table S6 — Associations between Machine-derived Oligodendroglioma Component (MOC) groups and gene copy number variations. P-values for (top row) enrichment, and (bottom row) depletion analysis of (left) genetic deletion (-2=homozygous deletion; -1=hemizygous deletion), (middle) no change (0=neutral/no change) and (right) amplification (2=high level amplification) within the three MOC groups were calculated using the right and left hypergeometric tails respectively. (DOC) [file pone.0081049.s011.doc]

**Table S6.** Associations between Machine-derived Oligodendroglioma Component (MOC) groups and gene copy number variations. P-values for (top row) enrichment, and (bottom row) depletion analysis of (left) genetic deletion (-2=homozygous deletion; -1=hemizygous deletion), (middle) no change (0=neutral/no change) and (right) amplification (2=high level amplification) within the three MOC groups were calculated using the right and left hypergeometric tails respectively.

|  | **MOC 0** | **MOC 1** | **MOC 2** |
| --- | --- | --- | --- |
| **CDK4** | 0.3836,0.6452,0.4107  0.6164,0.3548,0.5893 | 0.5215,0.4530,0.5298  0.4785,0.5470,0.4702 | 0.6055,0.3483,0.5751  0.3945,0.6517,0.4249 |
| **CDKN2A** | 0.5371,0.4629, N/A  0.4629,0.5371, N/A | 0.6665,0.3335, N/A  0.3335,0.6665, N/A | 0.1802,0.8198, N/A  0.8198,0.1802, N/A |
| **EGFR** | 0.2909,0.9000,0.1556  0.7091,0.1000,0.8444 | 0.6727,0.2341,0.6931  0.3273,0.7659,0.3069 | 0.5364,0.1561,0.8226  0.4636,0.8439,0.1774 |
| **EGLN2** | 0.2583,0.7417, N/A  0.7417,0.2583, N/A | 0.6917,0.3083, N/A  0.3083,0.6917, N/A | 0.5500,0.4500, N/A  0.4500,0.5500, N/A |
| **IDH1** | 0.5422,0.4578, N/A  0.4578,0.5422, N/A | 0.3735,0.6265, N/A  0.6265,0.3735, N/A | 0.5813,0.4187, N/A  0.4187,0.5813, N/A |
| **MDM2** | 0.3721,0.6486,0.4169  0.6279,0.3514,0.5831 | 0.5243,0.4010,0.5842  0.4757,0.5990,0.4158 | 0.6149,0.4421,0.4630  0.3851,0.5579,0.5370 |
| **NF1** | 0.1748,0.8252, N/A  0.8252,0.1748, N/A | 0.9463,**0.0537**, N/A  **0.0537**,0.9463, N/A | 0.1589,0.8411, N/A  0.8411,0.1589, N/A |
| **NOTCH2** | 0.4041,0.5959, N/A  0.5959,0.4041, N/A | 0.7599,0.2401, N/A  0.2401,0.7599, N/A | 0.2279,0.7721, N/A  0.7721,0.2279, N/A |
| **PDGFRA** | 0.3616,0.1134,0.9423  0.6384,0.8866,**0.0577** | 0.5439,0.5210,0.4403  0.4561,0.4790,0.5597 | 0.6067,0.9706,**0.0131**  0.3933,**0.0294**,0.9869 |
| **PTEN** | 0.3276,0.6724, N/A  0.6724,0.3276, N/A | 0.7332,0.2668, N/A  0.2668,0.7332, N/A | 0.4237,0.5763, N/A  0.5763,0.4237, N/A |
| **TP53** | 0.1508,0.8492, N/A  0.8492,0.1508, N/A | 0.9354,**0.0646**, N/A  **0.0646**,0.9354, N/A | 0.2299,0.7701, N/A  0.7701,0.2299, N/A |
