# Supplementary material for: Machine-Based Morphologic Analysis of Glioblastoma Using Whole-Slide Pathology Images Uncovers Clinically Relevant Molecular Correlates
Source: PLoS One. 2013 Nov 13;8(11):e81049. doi: 10.1371/journal.pone.0081049 (PMC3827469; doi:10.1371/journal.pone.0081049)
Supplement: Table S7 — Associations between Human-annotated Oligodendroglioma Component (HOC) groups and copy number variations. P-values for (top row) enrichment, and (bottom row) depletion analysis of (left) genetic deletion (-2=homozygous deletion; -1=hemizygous deletion), (middle) no change (0=neutral/no change) and (right) amplification (2=high level amplification) within the three HOC groups were calculated using the right and left hypergeometric tails respectively. (DOC) [file pone.0081049.s012.doc]

**Table S7.** Associations between Human-annotated Oligodendroglioma Component (HOC) groups and copy number variations. P-values for (top row) enrichment, and (bottom row) depletion analysis of (left) genetic deletion (-2=homozygous deletion; -1=hemizygous deletion), (middle) no change (0=neutral/no change) and (right) amplification (2=high level amplification) within the three HOC groups were calculated using the right and left hypergeometric tails respectively.

|  | **HOC 0** | | **HOC 1** | **HOC 2** | |
| --- | --- | --- | --- | --- | --- |
| **CDK4** | 0.1687,0.2901,0.8607  0.8313,0.7099,0.1393 | 0.7810,0.5667,0.2724  0.2190,0.4333,0.7276 | | | 0.5891,0.7681,0.1741  0.4109,0.2319,0.8259 |
| **CDKN2A** | 0.6063,0.3937, N/A  0.3937,0.6063, N/A | 0.3445,0.6555, N/A  0.6555,0.3445, N/A | | | 0.5845,0.4155, N/A  0.4155,0.5845, N/A |
| **EGFR** | 0.3000,0.8008,0.2762  0.7000,0.1992,0.7238 | 0.6636,0.1865,0.7506  0.3364,0.8135,0.2494 | | | 0.5364,0.4843,0.4852  0.4636,0.5157,0.5148 |
| **EGLN2** | 0.8583,0.1417, N/A  0.1417,0.8583, N/A | 0.0917,0.9083, N/A  0.9083,0.0917, N/A | | | 0.5500,0.4500, N/A  0.4500,0.5500, N/A |
| **IDH1** | 0.2429,0.7571, N/A  0.7571,0.2429, N/A | 0.7038,0.2962, N/A  0.2962,0.7038, N/A | | | 0.5701,0.4299, N/A  0.4299,0.5701, N/A |
| **MDM2** | 0.1646,0.6272,0.5933  0.8354,0.3728,0.4067 | 0.7789,0.1661,0.7041  0.2211,0.8339,0.2959 | | | 0.5997,0.8250,0.1165  0.4003,0.1750,0.8835 |
| **NF1** | 0.1773,0.8227, N/A  0.8227,0.1773, N/A | 0.6425,0.3575, N/A  0.3575,0.6425, N/A | | | 0.8078,0.1922, N/A  0.1922,0.8078, N/A |
| **NOTCH2** | 0.3595,0.6405, N/A  0.6405,0.3595, N/A | 0.8522,0.1478, N/A  0.1478,0.8522, N/A | | | 0.1960,0.8040, N/A  0.8040,0.1960, N/A |
| **PDGFRA** | 0.1753,0.0829,0.9819  0.8247,0.9171,**0.0181** | 0.7724,0.7654,0.1019  0.2276,0.2346,0.8981 | | | 0.5901,0.8914,**0.0655**  0.4099,0.1086,0.9345 |
| **PTEN** | 0.1014,0.8986, N/A  0.8986,0.1014, N/A | 0.9231,0.0769, N/A  0.0769,0.9231, N/A | | | 0.4899,0.5101, N/A  0.5101,0.4899, N/A |
| **TP53** | 0.0702,0.9298, N/A  0.9298,0.0702, N/A | 0.8061,0.1939, N/A  0.1939,0.8061, N/A | | | 0.8501,0.1499, N/A  0.1499,0.8501, N/A |
