# Supplementary material for: Machine-Based Morphologic Analysis of Glioblastoma Using Whole-Slide Pathology Images Uncovers Clinically Relevant Molecular Correlates
Source: PLoS One. 2013 Nov 13;8(11):e81049. doi: 10.1371/journal.pone.0081049 (PMC3827469; doi:10.1371/journal.pone.0081049)
Supplement: Table S8 — Associations between Machine-derived Oligodendroglioma Component (MOC) groups and human-annotated histology groups. P-values for (top row) enrichment, and (bottom row) depletion of pathologic ratings (left: absence, middle: presence, and right: abundance) within the three MOC groups were calculated using the right and left hypergeometric tails respectively. (DOC) [file pone.0081049.s013.doc]

**Table S8.** Associations between Machine-derived Oligodendroglioma Component (MOC) groups and human-annotated histology groups. P-values for (top row) enrichment, and (bottom row) depletion of pathologic ratings (left: absence, middle: presence, and right: abundance) within the three MOC groups were calculated using the right and left hypergeometric tails respectively.

|  | **MOC 0** | | **MOC 1** | **MOC 2** |
| --- | --- | --- | --- | --- |
| **Microvascular**  **Hyperplasia** | **0.0088**,0.9382,0.6840  0.9912,**0.0618**,0.3160 | 0.9916,0.1382,0.1524  **0.0084**,0.8618,0.8476 | | 0.5494,0.1804,0.8301  0.4506,0.8196,0.1699 |
| **Endothelial**  **Hyperplasia** | 0.6022,0.7606,0.1933  0.3978,0.2394,0.8067 | 0.2754,0.6345,0.4807  0.7246,0.3655,0.5193 | | 0.6818,**0.0152**,0.9699  0.3182,0.9848,**0.0301** |
| **Pseudopalisading**  **Necrosis** | 0.8583,**0.0306**,0.9180  0.1417,0.9694,0.0820 | 0.4154,0.8167,0.1184  0.5846,0.1833,0.8816 | | **0.0477**,0.9755,0.2969  0.9523,**0.0245**,0.7031 |
| **Zonal**  **Necrosis** | 0.2659,0.9246,0.1517  0.7341,**0.0754**,0.8483 | 0.7660,0.1800,0.6185  0.2340,0.8200,0.3815 | | 0.4250,0.1430,0.9276  0.5750,0.8570,**0.0724** |
| **Small**  **Cells** | 0.9821,0.2302,**0.0299**  **0.0179**,0.7698,0.9701 | **0.0842**,0.6249,0.9130  0.9158,0.3751,**0.0870** | | **0.0667**,0.7576,0.8421  0.9333,0.2424,0.1579 |
| **Gemistocytes** | 0.4609,0.4390,0.6459  0.5391,0.5610,0.3541 | 0.5383,0.7008,0.1898  0.4617,0.2992,0.8102 | | 0.5108,0.2428,0.8037  0.4892,0.7572,0.1963 |
| **Satllitosis** | 0.2255,0.7476,0.6174  0.7745,0.2524,0.3826 | 0.8282,0.2109,0.3217  0.1718,0.7891,0.6783 | | 0.4076,0.5494,0.5593  0.5924,0.4506,0.4407 |
| **Giant**  **Cells** | 0.6209,0.5908,0.1527  0.3791,0.4092,0.8473 | 0.5730,0.2728,0.7699  0.4270,0.7272,0.2301 | | 0.1804,0.7376,0.6600  0.8196,0.2624,0.3400 |
| **Epithelial**  **Metaplasia** | **0.0760**,0.7490,0.9124  0.9240,0.2510,**0.0876** | 0.9057,0.3672,**0.0534**  **0.0943**,0.6328,0.9466 | | 0.6480,0.2399,0.6124  0.3520,0.7601,0.3876 |
| **Sarcomatous**  **Metaplasia** | 0.7590,0.8230,**0.0179**  0.2410,0.1770,0.9821 | 0.3163,0.1898,0.9564  0.6837,0.8102,**0.0436** | | 0.3482,0.4050,0.7576  0.6518,0.5950,0.2424 |
| **Inflammation** | **0.0445,**0.8889,0.8015  0.9555,0.1111,0.1985 | 0.8463,0.2731,0.2361  0.1537,0.7269,0.7639 | | 0.9170,0.1094,0.3520  **0.0830**,0.8906,0.6480 |
| **Macrophages** | **0.0173**,0.9678,0.8087  0.9827,**0.0322**,0.1913 | 0.7891,0.3175,0.1609  0.2109,0.6825,0.8391 | | 0.9907,**0.0076**,0.5304  **0.0093**,0.9924,0.4696 |
| **Lymphocytes** | **0.0959**,0.8564,0.6922  0.9041,0.1436,0.3078 | 0.8103,0.2293,0.3743  0.1897,0.7707,0.6257 | | 0.8131,0.2428,0.3247  0.1869,0.7572,0.6753 |
| **Neutrophils** | 0.8843,0.1157, N/A  0.1157,0.8843, N/A | 0.1541,0.8459, N/A  0.8459,0.1541, N/A | | 0.4134,0.5866, N/A  0.5866,0.4134, N/A |
| **Cortex**  **Invasion** | 0.2007,0.7751,0.6174  0.7993,0.2249,0.3826 | 0.8734,0.1564,0.3217  0.1266,0.8436,0.6783 | | 0.31147,0.6520,0.5593  0.6853,0.3489,0.4407 |
| **White Matter Invasion** | **0.0913**,0.9472,0.1895  0.9069,**0.0528**,0.8105 | 0.8463,0.1005,0.7709  0.1537,0.8995,0.2291 | | 0.7441,0.2270,0.5593  0.2559,0.7730,0.4407 |
| **Mineralization** | 0.8823,0.1177, N/A  0.1177,0.8823, N/A | 0.2121,0.7879, N/A  0.7879,0.2121, N/A | | 0.2597,0.7403, N/A  0.7403,0.2579, N/A |
