# Supplementary material for: Machine-Based Morphologic Analysis of Glioblastoma Using Whole-Slide Pathology Images Uncovers Clinically Relevant Molecular Correlates
Source: PLoS One. 2013 Nov 13;8(11):e81049. doi: 10.1371/journal.pone.0081049 (PMC3827469; doi:10.1371/journal.pone.0081049)
Supplement: Table S9 — Associations of pathologic features shared by Human-annotated (HOC) and Machine-derived Oligodendroglioma Component (MOC) groups. We list all significant enrichment/depletion findings shared in HOC- and MOC-based correlative studies with pathologic ratings. Pathologic ratings 0, 1, 2 represent “absence”, “presence”, and “abundance”. (DOC) [file pone.0081049.s014.doc]

**Table S9. Associations of pathologic features shared by Human-annotated (HOC) and Machine-derived Oligodendroglioma Component (MOC) groups. We list all significant enrichment/depletion findings shared in HOC- and MOC-based correlative studies with pathologic ratings. Pathologic ratings 0, 1, 2 represent “absence”, “presence”, and “abundance”.**

| **Pathology Category** | **Enrichment / Depletion Status** |
| --- | --- |
| Microvascular hyperplasia | Patients with rating 0 are enriched in OC 0 group |
| Pseudopalisading necrosis | Patients with rating 1 are depleted in OC 2 group |
| Sarcomatous metaplasia | Patients with rating 2 are enriched in OC 0 group |
| Sarcomatous metaplasia | Patients with rating 2 are depleted in OC 1 group |
| White matter invasion | Patients with rating 1 are depleted in OC 0 group |
