# Supplementary material for: Machine-Based Morphologic Analysis of Glioblastoma Using Whole-Slide Pathology Images Uncovers Clinically Relevant Molecular Correlates
Source: PLoS One. 2013 Nov 13;8(11):e81049. doi: 10.1371/journal.pone.0081049 (PMC3827469; doi:10.1371/journal.pone.0081049)
Supplement: Text S1 — Endnote Library S1. (DOC) [file pone.0081049.s017.doc]

**Supporting Information**

**Glossary of Abbreviations**

TCGA: The Caner Genome Atlas: This is a publicly available dataset used for this study (https://tcga-data.nci.nih.gov/tcga/).

OC: Oligodendroglioma Component: This is the pathologic feature of interest to be measured both qualitatively by TCGA neuropathologists and quantitatively by computer-based analysis.

NS: Nuclear Score: This is a scalar assigned to each nucleus, ranging from 1 to 10, with 1 representing a classic oligodendroglioma and 10 a classic astrocytoma.

OCP (OC%): Oligodrendroglial Component Percentage: This is a measure of OC presented in each patient and calculated as the percentage of low NS nuclei in the combined set of low and high NS nuclei.

HOC: Human annotated Oligodendroglioma Component: This is a neuropathologist’s assessment of the degree of OC. Each TCGA glioblastoma was rated as absent (0), present (1+), or abundant (2+) by a panel of board certified neuropathologists. We use HOC $i$ to represent human-annotated patient population rated as $i$=0 for OC absent, 1 for OC present, and 2 for OC abundant.

MOC: Machine derived Oligodendroglioma Component: This is our computational assessment of the degree of OC. We clustered TCGA glioblastomas to three groups as counterparts to HOC groups. We use MOC $i$ to represent machine-derived patient population $i$=0 for OC absent, 1 for OC present, and 2 for OC abundant.

**Materials and Methods**

Image Partition and Computation Infrastructure

Whole-slide microscopic images were scanned with high resolution, making image analysis procedures intractable. A single whole-slide TCGA image can exceed 1GB in size. As a result, data structures and intermediate results retained during a whole slide image analysis cycle often exceed the main memory of a single machine. Moreover, the time for processing a whole slide image on a single machine is often too long for adoption. For these reasons, we partitioned whole slide images into non-overlapping 4096x4096-pixel tiles to accommodate image analysis requirements on a single machine and to enable parallel analysis. The choice of tile size represented a tradeoff between memory requirements and loss of microanatomy due to tiling. Large region sizes can cause analysis modules to exceed physical memory constraints. Conversely, smaller region sizes result in losing a larger proportion of nuclei on region boundaries for analysis.

Partitioning whole-slide images into tiles provides a straightforward approach to scale up the parallel image process within a large-scale, high-performance computer cluster environment where a large set of nodes executes jobs simultaneously. Our computational infrastructure configuration consists of seven Dell 1950 1U rack mount units, each configured with Dual Xeon E5420 CPUs running with four cores at 2.5Ghz for a total of eight cores per node. Each node has one job slot per core for a total of 56 jobs slots on the machine. Additionally, one node serves as the ``head node’’ where users can submit jobs to the system using Sun Grid Engine version 6.2u3 as its job scheduler.

Automated Pipeline for Analysis of Oligodendroglial Component (OC) in GBM

With the aim of quantitatively analyzing oligodendroglial and astrocytic morphologic components reflected by neoplastic nuclei within GBM tissue sections, we developed a complete workflow for high-throughput analysis of nuclear features. The workflow began with segmentation of nuclei from large-scale microscopy images for obtaining nuclei boundary coordinates. All nuclei present in digital slides were segmented regardless of type. To restrict analysis to neoplastic tissues, human reviewers annotated neoplastic regions within images with a Tablet device and only nuclei within those regions were selected for downstream analysis.

Segmentation of nuclei was followed by the computation of four complementary categories of nuclear features: morphometry, region texture, intensity, and gradient statistics. For optimal nuclear representation, we only used a subset of discriminative features for oligodendroglioma component analysis in the downstream experiments. The most discriminating feature subset consisted of features from all four categories.

Since diffuse gliomas can be viewed as mixtures of oligodrengroglioma, astrocytoma, and intermediate morphology variations, we developed a computer-based method to reproducibly quantify tumor cell components. We defined the Nuclear Score (NS), scaled from 1 to 10, with 1 representing a classic oligodendroglioma and 10 a classic astrocytoma. Values between 1 and 10 represent nuclei exhibiting nuclear features across the oligodrengroglioma-astrocytoma continuum. With this definition, each nucleus was assigned an NS. This approach enabled us to analyze the full spectrum of glioma nuclei quantitatively. Given the NS definition for Oligodendroglial Component (OC) recognition, we applied the regression analysis with use of the generalized linear regression function to NS computation.

To facilitate collection of NS from human annotators for the purpose of regression model training, a user-friendly graphical interface was developed. Nuclei from a separate set of images archived at Emory University Hospital were pre-segmented, with associated features pre-computed by computer algorithms. Users could click on a nucleus of interest and chose the corresponding NS in the feature Table. At the end of training, results were exported and saved. All meta-data, including human annotations, nuclear boundary coordinates, calculated features, computed NS, and analysis method information were then recorded in and managed by a central database using the in-house developed Pathology Analytical and Imaging Standards (PAIS) model, which was designed to provide a flexible, efficient, and semantically enabled data structure for pathology image analysis and characterization.

For each patient, we then aggregated NSs to a single scalar that was defined as Oligodendroglial Component Percentage (OC%) calculated by counts of neoplastic nuclei within the low and high NS intervals. Additionally, we used the unsupervised K-means clustering algorithm with 10000 seed points to reliably partition patients into three Machine-derived Oligodendroglioma Component (MOC) groups on the basis of their OC%s and compared with analogous groupings based on annotations by TCGA neuropathologists.

Nuclei Segmentation

Nuclear analysis began with segmentation. To accommodate the demanding image resolution, we partitioned whole slide images into non-overlapping 4096x4096-pixel tiles. The first step in the segmentation module involved recognition of non-tissue and red blood cell regions. The percent area occupied either by non-tissue spaces or red blood cells was computed to determine whether a given tile contains sufficient tumor tissue for analysis. Only the tissue areas in valid tiles were further processed. The segmentation method was designed to be computationally inexpensive, yet no simpler than required for accuracy and robustness. We focused on extracting foreground objects of interest (nuclei) from background signals that displayed large variations. As most issues in microscopy image analysis algorithms arise from variations in image intensity, color, texture, and data scale, we used a fast hybrid grayscale reconstruction algorithm to normalize background regions degraded by artifacts introduced from the tissue preparation and scanning stage. This step allowed us to separate foreground from normalized background with a simple threshold-based mechanism, which is critical for segmenting efficiently and accommodating the identification of nuclei with distinct features. Overlapped nuclei were separated using the watershed technique. In post-processing, detected objects not satisfying either area or shape constraints were filtered out.

Nuclei Regression Analysis

We used regression analysis for NS computation. The generalized linear regression function was used because linear models are straightforward and most appropriate for revealing the dominant patterns between NS and nuclear features. Linear models are also less subject to over-fitting than non-linear ones, as they do not take into account the sensitive effects of cross-terms. However, it is established that linear least-square estimates are heavily subject to both outliers and heavy-tailed error distribution. Therefore, we used the Iteratively Reweighted Least-Square criterion (IRLS) to mitigate the influence from outlier data.

In this study, the generalized linear regression function defined in the following format was used:

(S1)

where represents the i-th feature and $\varepsilon$ is a normally distributed random variable representing the error term. The vector of predictors is , while y is the response. $\left[\begin{matrix} \beta_{0} & \beta_{1} & ... & \beta_{C} \end{matrix}\right]$ is a vector of C+1 coefficients to be determined. With this approach, we now aim to minimize the following cost function:

$E(\beta)=\sum_{i=1}^{N} f(w_{i})=\sum_{i=1}^{N} f(y_{i}-x_{i}^{T}\beta)$ (S2)

where$f\left(\bullet\right)$ is a function that evaluates the contribution of each residual to the overall cost function. In our study, we chose $f\left(\bullet\right)$to be the bi-square objective function, as the associated weight function decreases sharply when residual departs off 0. The best parameters minimizing the aforementioned cost function can be solved by equating to zero the derivative of the cost function with respect to the parameter set:

$\beta=\left(X^{T}WX\right)^{-1}X^{T}WY$ (S3)

where is a diagonal matrix determined by residuals and $w(e)=f'(e)/e$.

It is noticed that W is a matrix determined by residuals. Nevertheless, residuals depend on the estimated parameters and the parameters rely on the weight functions in turn. As a result, we consider the parameters as functions of time stamp t and can be estimated using iterative computations:

(S4)

The parameters estimated at time stamp t are determined using the weights estimated at time (t-1) that in turn are computed from the estimated parameters at time (t-1). As a result, an iterative computation process can gradually yield a stabilized coefficient vector after a few iterations. Using stabilized values of these coefficients, we calculated the NS for each identified nucleus in whole-slide images.
